# Improving Alzheimer’s Disease and Parkinson’s Disease in Rats with Nanoemulsion and Byproducts Prepared from Cinnamon Leaves

**DOI:** 10.3390/pharmaceutics17091200

**Published:** 2025-09-15

**Authors:** Bing-Huei Chen, Chen-Te Jen, Chia-Chuan Wang, Min-Hsiung Pan

**Affiliations:** 1 Department of Food Science, Fu Jen Catholic University, New Taipei City 242062, Taiwan; 2 Graduate Institute of Food Science, National Taiwan University, Taipei City 106319, Taiwan; d11641002@ntu.edu.tw; 3 School of Medicine, College of Medicine, Fu Jen Catholic University, New Taipei City 242062, Taiwan; 050814@mail.fju.edu.tw

**Keywords:** cinnamon leaves, nanoemulsion, cinnamaldehyde, UPLC-MS/MS, Alzheimer’s disease, Parkinson’s disease, Morris maize water test

## Abstract

**Background/Objectives**: Cinnamon leaves, an important source of the functional compound cinnamaldehyde (CA), have been shown to be effective in improving type II diabetes and Parkinson’s disease (PD) in rats following the incorporation of cinnamon leaf extract into a nanoemulsion. However, the effect of a cinnamon leaf extract nanoemulsion (CLEN) on improving Alzheimer’s disease (AD), the most prevalent type of dementia, remains unexplored. The objectives of this study were to determine functional compounds in cinnamon leaves by UPLC-MS/MS, followed by the preparation of a nanoemulsion and its byproducts to study their effects on AD and PD in rats. **Methods**: Oven-dried (60 °C for 2 h) cinnamon leaf powder and hydrosol, obtained by steam distillation of cinnamon leaf powder, were stored at 4 °C. After determination of basic composition (crude protein, crude fat, carbohydrate, moisture and ash) of cinnamon leaf powder, it was extracted with 80% ethanol with sonication at 60 °C for 2 h and analyzed for bioactive compounds by UPLC-MS/MS. Then, the CLEN was prepared by mixing cinnamon leaf extract rich in CA with lecithin, soybean oil, tween 80 and ethanol in an optimal ratio, followed by evaporation to form thin-film and redissolving in deionized water. For characterization, mean particle size, polydispersity index (PDI), zeta potential, encapsulation efficiency, and surface morphology were determined. Animal experiments were done by dividing 90 male rats into 10 groups (n = 9), with groups 2–8 being subjected to mini-osmotic pump implantation surgery in brain to infuse Amyloid-beta 40 (Aβ40) solution in groups 2–8 for induction of AD, while groups 9 and 10 were pre-fed respectively with cinnamon powder in water (0.5 g/10 mL) and in hydrosol for 4 weeks, followed by induction of AD as shown above. Different treatments for a period of 4 weeks included groups 1–9, with group 1 (control) and group 2 feeding with sterilized water, while groups 3, 4 and 5 were fed respectively with high (90 mg/kg), medium (60 mg/kg) and low (30 mg/kg) doses of cinnamon leaf extracts, groups 6, 7 and 8 fed respectively with high (90 mg/kg), medium (60 mg/kg) and low (30 mg/kg) doses of nanoemulsions, groups 9 and 10 fed respectively with 10 mL/kg of cinnamon powder in water and hydrosol (0.5 g/10 mL). Morris water maze test was conducted to determine short-term memory, long-term memory and space probing of rats. After sacrificing of rats, brain and liver tissues were collected for determination of Aβ40, BACE1 and 8-oxodG in hippocampi, and AchE and malondialdehyde (MDA) in cortices, antioxidant enzymes (SOD, CAT, GSH-Px) and MDA in both cortices and livers, and dopamine in brain striata by using commercial kits. **Results**: The results showed that the highest level of CA (18,250.7 μg/g) was in the cinnamon leaf powder. The CLEN was prepared successfully, with an average particle size of 17.1 nm, a polydispersity index of 0.236, a zeta potential of −42.68 mV, and high stability over a 90-day storage period at 4 °C. The Morris water maze test revealed that the CLEN treatment was the most effective in improving short-term memory, long-term memory, and spatial probe test results in AD rats, followed by the cinnamon leaf extract (CLE), powder in hydrosol (PH), and powder in water (PW). Additionally, both CLEN and CLE treatments indicated a dose-dependent improvement in AD rats, while PH and PW were effective in preventing AD occurrence. Furthermore, AD occurrence accompanied by PD development was demonstrated in this study. With the exception of the induction group, declines in Aβ40, BACE1, and 8-oxodG in the hippocampi and AchE and MDA in the cortices of rats were observed for all the treatments, with the high-dose CLEN (90 mg/kg bw) exhibiting the highest efficiency. The antioxidant enzyme activity, including that of SOD, CAT, and GSH-Px, in the cortices of rats increased. In addition, dopamine content, a vital index of PD, was increased in the striata of rats, accompanied by elevations in SOD, CAT, and GSH-Px and decreased MDA in rat livers. **Conclusions**: These outcomes suggest that the CLEN possesses significant potential for formulation into a functional food or botanical drug for the prevention and treatment of AD and/or PD in the future.

## 1. Introduction

Alzheimer’s disease (AD), discovered by German clinical psychiatrist Alzheimer Alois in 1906, has become the most prevalent neurodegenerative disease worldwide. According to a report from Alzheimer’s Disease International, a total of 55 million people worldwide suffered from dementia in 2019, and this number is expected to climb to 78 and 139 million in 2030 and 2050, respectively, with AD accounting for approximately 60% of cases [1]. AD is characterized by the presence of extracellular senile plaques and intracellular neurofibrillary tangles, with the former being mainly composed of amyloid β (Aβ), and the latter being made up of hyperphosphorylated tau proteins [2]. It is generally recognized that AD occurs as a result of the gradual and progressive loss of neuronal function, ultimately leading to neuronal cell death [3]. Although numerous studies have elucidated possible causes of and treatments for AD, there is no known cure for AD.

Based on published reports, there are many critical features of AD, including the elevation of neuroinflammation and oxidative stress, the impairment of mitochondria function, autophagy dysfunction, the excessive accumulation of iron, synapse loss, and the malfunction of neurotransmitters [2,3]. Among these features, oxidative stress has been shown to play a vital role in the AD progression [4]. Thus, it is possible to ameliorate AD via a reduction in oxidative stress through the incorporation of antioxidants such as pongamol, a natural flavonoid isolated from leguminous plants [5]. Moreover, antioxidants such as polyphenol, astaxanthin, and isoflavone have been shown to be effective in reducing Aß deposition, regulating signal pathways, retarding the phosphorylation of tau protein, and protecting neuron integrity through reduction in oxidative stress [6,7]. However, these antioxidants often suffer low bioavailability under gastrointestinal conditions, thereby affecting therapeutic efficiency in vivo. To address this issue, the possible therapeutic effects of natural products against AD, using nanotechnology for the encapsulation of unstable bioactive compounds, have gained increasing attention.

Parkinson’s disease (PD), another neurodegenerative disorder, is mainly caused by the loss of dopamine-producing neurons in the substantia nigra and basal ganglia striatum and the subsequent accumulation of α-synuclein and the formation of Lewy bodies, eventually leading to motor symptoms such as muscle rigidity, tremors, and slow movement [8]. Like AD, PD also has many critical features, including oxidative stress, mitochondria dysfunction, chronic inflammation, insulin resistance, the generation of advanced glycation end products, and autophagy function impairment [8]. As both AD and PD share common neuropathologic features, it is feasible to develop a natural product for the simultaneous treatment of AD and PD. Furthermore, the current drugs used for AD treatment include acetylcholinesterase inhibitors (AChEIs), N-methyl-D-aspartate receptor (NDMAR) antagonist, and anti-amyloid beta monoclonal antibodies. However, many side effects, such as headache, dizziness, vomiting, diarrhea, nausea, insomnia, confusion, and amyloid-related imaging abnormalities, have been observed [9,10,11]. Similar side effects were also found for drugs for PD treatment, including levodopa, dopamine agonists, and enzyme inhibitors [12]. As no drugs are currently available for the dual therapy of AD and PD, the development of a plant-derived nanoproduct for the treatment of both diseases with minimal side effects is pivotal.

Cinnamon, a traditional herbal medicine that has been used for thousands of years and has been determined to be safe by the United States Food and Drug Administration, has been reported to possess many positive biological functions, such as antioxidation, anti-cancer, anti-inflammation, anti-type II diabetes, and anti-PD functions in both in vitro and in vivo studies [8,13,14]. The presence of certain functional compounds in cinnamon, such as cinnamaldehyde (CA) and cinnamic acid, may be responsible for the biological effects observed [8]. However, two cinnamon species, *Cinnamomum cassia* Blume and *Cinnamomum zeylanicu* Brey, have been shown to contain a high level of carcinogenic coumarin in cinnamon bark [8]. But for the leaves of a cinnamon species (*C. osmophloeum*) grown in Taiwan, coumarin was present only at trace levels [15]; thus, cinnamon leaves can be used as a safe material for the production of cinnamon-derived products for ameliorating chronic diseases. In addition, compared to cinnamon bark, which needs to be stripped from trees using a knife, cinnamon leaves are more environmentally friendly.

In a previous study, Huang and Chen [14] developed a nanoemulsion system consisting of cinnamon leaf extract (CLE), lecithin, Tween 80, soybean oil, and deionized water, with an average particle size 36.58 nm, and demonstrated that a 60 mg/kg dose of cinnamon leaf extract nanoemulsion (CLEN) was the most effective in alleviating streptozotocin-induced type II diabetes in rats. As several type II diabetes medications have shown positive effects on PD, a similar nanoemulsion system with an average particle size of 30.1 nm was prepared by Wang et al. [8], who reported that a 60 mg/kg dose of CLEN was the most effective in ameliorating rotenone-induced PD in rats through the elevation of dopamine and tyrosine hydroxylase levels, with a concomitant decline in α-synuclein content in the rat striatum and the alleviation of oxidative stress via an increase in antioxidant enzyme activities, including superoxide dismutase (SOD), catalase (CAT), and glutathione peroxidase (GSH-Px), as well as a decrease in the malondialdehyde (MDA) content in the rat midbrain. This finding implies that CLEN could restore dopaminergic signaling to trigger a cascade of cellular events for improving cognitive and motor functions in PD rats. However, an elevated level of α-synuclein has been shown to enhance the harmful effects of tau protein and to interact with Aβ-amyloid to promote AD neurodegeneration and cognitive decline [16]. Consequently, a close association has been shown to exist between type II diabetes and PD, type II diabetes and AD, and PD and AD [17,18,19]. Type II diabetes, caused by insulin resistance and subsequent low-level insulin secretion, can lead to long-term hyperglycemia, a primary factor for neurological diseases such as PD and AD [20].

Moreover, AD typically progresses more rapidly and primarily impacts the hippocampus and cerebral cortex, with cognitive decline being the core feature, while PD has a slow progression and mainly affects the substantia nigra and basal ganglia striatum, with motor symptoms worsening over time [8,21]. Interestingly, a significant percentage of AD patients possess extrapyramidal symptoms, while many PD patients develop dementia [21]. Thus, the simultaneous amelioration of the neuropathologic features of both AD and PD is feasible through development of an optimal nanosystem containing diversified compounds from cinnamon leaves, i.e., both surfactants and antioxidants.

Principally, the blood–brain barrier (BBB) is designed to protect the brain from harmful substances in the bloodstream; however, it also presents a challenge for drug delivery to the brain. Accumulating evidence has shown that BBB dysfunction can be an early biomarker of AD as the BBB is responsible for the clearance of 80–85% of AD-related forms of amyloid-β in the brain [22]. Additionally, BBB dysfunction can be caused by blood vessel impairment, leading to neuronal dysfunction [23]. Therefore, developing a nanosystem containing multiple bioactive compounds of a lipophilic nature for the simultaneous crossing of the BBB via passive diffusion while improving vascular function for the treatment of AD and PD is imperative. In a recent study, an improvement in type II diabetes and vascular function in rats upon the administration of a nanoemulsion was reported by Huang and Chen [14]. However, the effect of this nanoemulsion on alleviating AD remains unexplored.

It is worth pointing out that this is the first study to show the possible treatment of AD and PD in rats simultaneously by preparing a nanoemulsion system from cinnamon leaves. Unlike other tests for type II diabetes and PD, in this study, we used the Morris water maze test to verify the improvement of short-term memory, long-term memory, and spatial probing in AD rats, followed by the measurement of various biochemical parameters, including acetylcholinesterase (AchE) in rat cortices; Aβ40, β-secretase (BACE1), and 8-hydroxy-2′-deoxyguanosine (8-oxodG) in rat hippocampi; dopamine in rat brain striata; and SOD, CAT, GSH-Px, and MDA in rat cortices and livers, for the elucidation of the possible mechanisms involved in AD progression. In addition, the preventive effects of cinnamon leaf powder in hydrosol or water with respect to AD or PD in rats were assessed, while the possibility of AD occurrence concomitant with PD development was demonstrated for the induction treatment. This study is intended to form a basis for the possible future development of a botanic drug for the prevention and treatment of AD and PD, using cinnamon leaves as a raw material.

## 2. Material and Methods

### 2.1. Processing of C. osmophloeum Leaves (Cinnamon Leaves) and Hydrosol

The cinnamon leaves, harvested from Taitung county (Taiwan) in October 2023, were supplied by Tou-Fu Co. (Taipei, Taiwan). About 1 kg of leaves were transported to our laboratory on the same day and washed for subsequent oven-drying at 60 °C for 2 h, as a high yield of functional compounds in cinnamon leaves was found under this drying condition [14]. Then, dried cinnamon leaves were ground into a powder, with a total amount of 540 g being obtained. For the preparation of hydrosol by steam distillation, a total of about 8 kg of cinnamon leaves was collected and distilled with pure water (50 L) at 100 °C for 3 h to obtain the H_2_O-soluble fraction (hydrosol), containing 97% water, with a total volume of 30 L [8]. Then, hydrosols were stored at 4 °C for further use.

### 2.2. Proximate Analysis of Cinnamon Leaves

The proximate analysis of both fresh and dried leaves, including crude protein, crude fat, moisture, and ash, was undertaken in duplicate using the standard procedures recommended by the Chinese National Standards (CNS) of the Republic of China (Taipei, Taiwan).

### 2.3. Determination of Functional Compounds in Cinnamon Leaves by UPLC-MS/MS

Functional compounds in cinnamon leaves were extracted based on a method reported by Wang et al. [8]. In brief, 1 g of cinnamon leaf powder was mixed with 30 mL of 80% ethanol, followed by sonication for 2 h at 60 °C and centrifugation for 20 min at 25 °C (4000 rpm). Following the repetition of this step three times, the supernatants were pooled for subsequent filtration through a filter paper and evaporation under nitrogen gas. Next, the residue was dissolved in 80% ethanol (10 mL) to obtain crude CLE and then stored at 4 °C for subsequent experiments. For UPLC-MS/MS analysis of functional compounds in cinnamon leaves, the CLE was filtered through a 0.22 μm membrane filter. But hydrosol was directly analyzed by UPLC-MS/MS, eliminating the extraction process.

A Luna Omega C18 column (100 × 2.1 mm ID, 1.6 μm particle size) with a mobile phase of 0.025% acetic acid in water (A) and 0.025% acetic acid in methanol (B) in gradient mode was used for UPLC-MS/MS: 83%A and 17% B in the beginning, changed to 80% A and 20% B at 1 min, 60% A and 40% B at 5 min, 45% A and 55% B at 10 min, 1% A and 99% B at 14 min, and then resuming the initial ratio. A total of 15 functional compounds, namely, eugenol, trans-cinnamic acid, kaempferol-3-β-D-glucopyranoside, kaempferol, CA, cinnamyl alcohol, quercetin, quercetin-3-O-galactoside, quercetin-3-O-glucoside, rutin, benzoic acid, coumarin, p-coumaric acid, caffeic acid, and 5-O-caffeoylquinic acid, were separated within 12 min, with a column temperature of 30 °C and a flow rate of 0.3 mL/min. However, both quercetin-3-O-glucoside (peak 8) and quercetin-3-O-galactoside (peak 7) overlapped. A multiple reaction monitoring (MRM) mode involving negative ion mode was used for detection [14]. The identification of each functional compound in cinnamon leaf powder and hydrosol was performed by comparing the mass spectra and retention time of sample peaks on the UPLC chromatogram with those of the standards.

For quantitation by UPLC-MS/MS, the standard curve of each functional compound shown above was prepared using 8 concentrations, including 0.01, 0.02, 0.05, 0.1, 0.2, 0.3, 0.4, and 0.5 µg/g, by plotting concentration against peak area, with the linear regression equation of each standard being used to calculate the amount of each functional compound present in cinnamon leaf powder and hydrosol, as reported by Wang et al. [8]. However, it should be pointed out that the method of validation was not performed in this study as a previous study had already demonstrated the high accuracy and precision of this extraction method [14]. In addition, to verify the reliability of label-free quantitation, out of the 8 CA standard concentrations (0.01, 0.02, 0.05, 0.1, 0.2, 0.3, 0.4, and 0.5 µg/g) used for preparation of the standard curve for CA quantitation, 3 standard concentrations (one each for low, medium, and high concentrations), including 0.02, 0.2, and 0.5 µg/g, were analyzed to verify the UPLC-MS/MS response during every batch of CA analysis.

### 2.4. Preparation of Cinnamon Leaf Extract Nanoemulsion (CLEN)

Initially, a portion of cinnamon leaf powder (200 g) was collected and mixed with 80% ethanol (500 mL), followed by mixing thoroughly for 1 h and collecting 20 mL for subsequent nanoemulsion preparation. An optimal amount and ratio of lecithin (0.2 g, 1%), soybean oil (0.1 g, 1%), and Tween 80 (0.6 g, 6%) was used and stirred completely, followed by adding CLE (20 mL) in a round-bottom flask, mixing thoroughly and evaporating under vacuum to form a thin film. Next, deionized water (9.1 g, 91%) was added and mixed completely, after which this solution was subjected to ultrasonication for 30 min to obtain CLEN (10 mL), containing 10,000 ppm CA, which was stored at 4 °C for further use.

### 2.5. Characterization of CLEN

Both the average particle size and polydispersity index (PDI) of the CLEN were measured using a dynamic light scattering instrument by diluting CLEN with KH_2_PO_4_ buffer (25 mM, pH 5.5) 50 times, filtering through a 0.22 μm polyvinylidene difluoride (PVDF) filter, and pouring into a cuvette for analysis. For zeta potential determination, a CLEN sample (100 μL) was diluted with deionized water 200 times for measurement at 25 °C. Additionally, both the shape and average particle size of the CLEN were measured using a transmission electron microscope (TEM) by diluting with deionized water 50 times, dropping 20 μL on a carbon-coated copper grid (90 s), and removing the excess sample with a filter paper. The sample was further negatively stained with 20 µL of phosphotungstic acid (30 s), followed by removing the excess stain with a filter paper and drying overnight in an oven for imaging at 120 kV.

Additionally, the reproducibility of the CLEN preparation was evaluated by determining the relative standard deviation (RSD) of intra-day and inter-day variability in the particle size, PDI, and zeta potential, with the CLEN being prepared in the morning, afternoon, and evening of the same day with three replicates each for the former and prepared on three consecutive days with three replicates each day for the latter.

### 2.6. Encapsulation Efficiency of CA in CLEN

Initially, a CLEN sample (100 μL) was mixed with n-hexane (400 μL) for shaking to dissolve free CA in the upper n-hexane layer for collection, while 100 μL of the same CLEN was mixed with ethanol (400 μL) for ultrasonication for 2 h to release total CA. Following HPLC analysis, using the same separation condition as described above, with UV detection at 280 nm, the encapsulation efficiency of CA was obtained using a formula described by Wang et al. [8]. For method validation, the CA standard (10 ppm) was added to the CLEN (100 µL), followed by mixing with n-hexane (400 µL) for shaking to dissolve and collect free CA in the upper layer, while 100 µL of the same CLEN containing the CA standard at 10 ppm was mixed with ethanol (400 µL) and ultrasonicated for 2 h to release total CA. The recovery data of free CA and total CA were calculated based on the ratio of free CA and total CA after HPLC to that before HPLC. The intra-day variability was carried out by determining free CA and total CA contents in the morning, afternoon, and evening on the same day, with three replicates each, while the inter-day variability was conducted by analyzing free CA and total CA in the morning, afternoon, and evening, each on the first, second, and third day, for a total of nine analyses. All the samples were filtered through a 0.22 µm PVDF filter and analyzed by HPLC with UV detection at 280 nm.

### 2.7. Animal Experiment

All animal experiments followed the standard experimental animal operation procedures and were approved by the Institutional Animal Care and Use Committee (IACUC) of Fu Jen University (New Taipei City, Taiwan). A total of 90 Wistar male rats, 8 weeks old and weighing about 250 g each, were procured from the National Experimental Animal Center of Taiwan (Taipei, Taiwan). All rats were raised in the Fu Jen University Experimental Animal Center at a temperature of 21 ± 2 °C, a relative humidity of 55% ± 10%, and an illumination cycle of 12 h. Both standard rodent chow diet and water were provided ad libitum during the experiment. Additionally, environmental conditions, including air quality, temperature, humidity, lighting, and noise levels, were standardized across all cages throughout the experiment, along with environmental enrichment in the form of wooden sticks and bedding materials to promote natural behavior and reduce stress. One rat was considered as one experimental unit, and the rats were randomly assigned to individual standard polycarbonate cages with stainless steel wire lids (one rat per cage) by the animal center personnel for subsequent numbering, with the control group being first, followed by the induction group and treatment groups. Both the position and the numbering order of the cages were maintained in an open rack, and all researchers involved in this study were informed of group allocation and the order of the cages during different stages of the experiment, including allocation, conduct of experiment, outcome assessment, and data analyses, to minimize potential confounders on the experimental outcome. The overall health of the rats was monitored once in two days by the animal center personnel. To reduce handling stress, rats were gently grasped by the tail and supported with the opposite hand when removed from the cage and subsequently calmed by gentle stroking before feeding. In addition, apparent signs of pain in rats, including vocalization, aggressive or defensive behavior, social withdrawal, or self-isolation, were effectively managed with subcutaneous injection of ketoprofen (2–3 mg/kg). It was also established that rats would be euthanized by CO_2_ inhalation if humane endpoints were encountered, including rapid weight loss, severe weakness with an inability to eat/drink, paralysis, and visible damage to vital organs. However, no expected or unexpected adverse events occurred during the study.

After adaptation for two weeks, with each rat weighing at about 300 g, 90 rats were divided into 10 groups of 9 rats each, in which groups 2–8 were subjected to brain pump implantation surgery (mini-osmotic pump, model 2004, Alzet Corporation, Palo Alto, CA, USA), while groups 9 and 10 were pre-fed with cinnamon powder dissolved in water and hydrosol, respectively, for 4 weeks before brain pump implantation surgery started. Although, typically, 8 rats are used per group, one additional rat per group was used as this study involved surgical procedures. Moreover, a criterion was set to exclude rats that died during the experiment. However, as no rats died in this study, all data analyses were conducted with 9 rats per group. The surgical operation was conducted following a report by Lin et al. [24] with a slight modification, with a brain infusion rate of 0.28 μL/h (6.72 μL per day for 28 days), an internal volume of 234 μL, a hose (2–3 cm long, internal volume of 80 μL), and a brain infusion kit II (3–5 mm, Alzet©). Rats were fixed and oriented by using a stereotaxic instrument for the subsequent infusion of amyloid β protein-40 (Aβ40) into brains for the induction of AD, with the Aβ40 solution being dissolved in 35% acetonitrile/0.1% trifluoroacetic acid (pH 2.0). Then, both the Aβ40 solution and the blank solution without Aβ40 were injected into an Alzet mini osmotic pump, separately connected to a brain infusion kit II so that the input tubes and heads were filled with Aβ40 solution. Following the anaesthetization of rats by intraperitoneal injection with sodium pentobarbital (50 mg/kg), rats were fixed on a stereotaxic instrument for the orientation of the left brains, with the skull bregma serving as the center for drilling holes with a tooth drill at the following position: vertical axis, 0.8 mm; horizontal axis, 1.4 mm. Next, a brain infusion kit II was implanted into the brain with a 4.0 mm depth from the skull, followed by fixing the input needle heads on the skull via bioadhesives. Then, Alzet mini osmotic pumps were placed on the back of the neck, and the wound was sutured, followed by a coating with a Spersin ointment containing neomycin sulfate (5 mg), bacitracin zinc (400 U), and polymyxin B sulfate (5000 U), and the rats were returned to the cage for care.

### 2.8. Morris Water Maze Test

The Morris water maze test was conducted in a circular pool with a diameter of 160 cm and a height of 60 cm, which was located in a dark room. A round and movable rest platform (escape platform) with a diameter of 12 cm and a height of 30 cm was located in the pool, and water was added to a level of 33 cm before the experiment started. The pool was divided into four quadrants, i.e., zones I, II, III, and IV, and four objects with different shapes (square, triangle, rhombus, round) were placed at the borderline between two quadrants to assist in rats’ spatial cognition. Meanwhile, a digital camera was erected above the middle point of the pool to record the swimming path of rats for subsequent analysis with video tracking software.

For the working memory task (i.e., the short-term memory test), the rest platform was placed in a quadrant (i.e., zones I, II, III, or IV) every day, with training 5 times each day for 3 days. Then rats were allowed to enter the pool from any of the other 3 quadrants randomly for 120 s. If rats found the rest platform within 120 s, rats were allowed to rest for 30 s and returned to their cages for a further rest of 60 s. Conversely, if rats could not find the rest platform within 120 s, rats were guided to the rest platform for 30 s and returned to cage for 60 s for next training. Finally, the mean results from the second to fifth trials of the 3rd day were calculated and analyzed statistically, except for the first trial, which was considered as cognitive training.

For the reference memory task (i.e., the long-term memory test), the rest platform was fixed at the first quadrant the next day following completion of the working memory task. Then, the rats were allowed to enter the pool from any of the other three quadrants for training 4 times every day with 120 s each. If rats found the rest platform within 120 s, they were allowed to rest for 30 s on the platform and returned to their cages for a further rest of 30 min for the next trial. If rats could not find the rest platform within 120 s, they were placed on the platform for 30 s and then returned to cages for 30 min for next trial. Only the results of the third day were calculated and statistically analyzed, as the first two days were mainly used for practice.

For the probe test, the rest platform was removed from the pool the next day following completion of the reference memory task. Then, the rats were allowed to enter the pool from the second quadrant for swimming for 180 s, with the swimming path and time spent at the first quadrant being recorded. Additionally, the wandering time in the target area (first quadrant), edge touching times, and time spent in the edge area were monitored and recorded.

A total of 10 animal treatments are shown below with the CLE being concentrated to remove ethanol and dissolve in sterilized water prior to animal experiment.

1. Control group (N): Fed with sterilized water.
2. Induction group (I) (induced with Aβ40 solution): Fed with sterilized water.
3. High-dose CLE (HE): Induced with Aß40 solution and fed with 90 mg/kg HE for 4 weeks.
4. Medium-dose CLE (ME): Induced with Aß40 solution and fed with 60 mg/kg ME for 4 weeks.
5. Low-dose CLE (LE): Induced with Aß40 solution and fed with 30 mg/kg LE for 4 weeks.
6. High-dose CLEN (HN): Induced with Aß40 solution and fed with 90 mg/kg HN for 4 weeks.
7. Medium-dose CLEN (MN): Induced with Aß40 solution and fed with 60 mg/kg MN for 4 weeks.
8. Low-dose CLEN (LN): Induced with Aß40 solution and fed with 30 mg/kg LN for 4 weeks.
9. Pre-fed cinnamon powder in water (0.5 g/10 mL) (PW) for 4 weeks: Induced with Aß40 solution and fed with 10 mL/kg for 4 weeks.
10. Pre-fed cinnamon powder in hydrosol (0.5 g/10 mL) (PH) for 4 weeks: Induced with Aß40 solution and fed with 10 mL/kg for 4 weeks.

**Figure 1:**
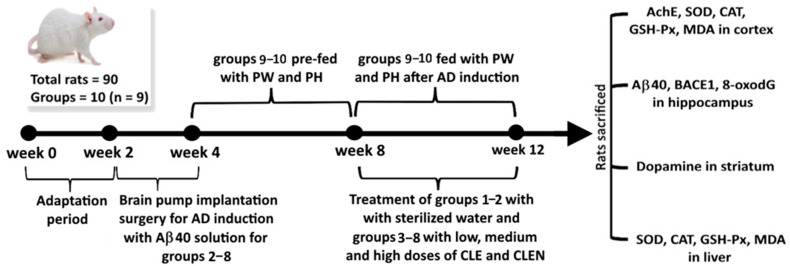
A schematic diagram illustrating the study design of animal experiments. AD, Alzheimer’s disease; CLE, cinnamon leaf extract; CLEN, cinnamon leaf extract nanoemulsion; PW, cinnamon powder in water; PH, cinnamon powder in hydrosol; AchE, acetylcholinesterase; SOD, superoxide dismutase; CAT, catalase; GSH-Px, glutathione peroxidase; MDA, malondialdehyde; Aβ40, amyloid beta 40; BACE1, β-secretase; 8-oxodG, 8-hydroxy-2′-deoxyguanosine.

After completion of the animal experiment, rats were anesthetized by carbon dioxide inhalation, sacrificed, and then brain tissues, including the cortex, hippocampus, and corpus striatum, as well as liver tissues, were collected for further analysis. A schematic diagram illustrating the study design of animal experiments is shown in Figure 1.

### 2.9. Measurement of Biochemical Parameters in the Rat Brain

Following the completion of the animal experiments, the rats were sacrificed using CO_2_, and both liver and brain tissues were collected. The brain tissue was divided into three parts, including the cortex, hippocampus, and corpus striatum. Following tissue removal, samples were rinsed with PBS buffer (0.01 M, pH 7.4) to remove blood and stored at −80 °C. For pretreatment, tissues were weighed and diluted 1:10 with PBS while being maintained in an ice bath, followed by homogenization, centrifugation (5000× *g*) for 5 min, and collection of the supernatant for storage at −20 °C until use.

Next, protein quantification was performed using the bicinchoninic acid (BCA) protein assay with a Visual Protein BC03-500 kit (Taipei, Taiwan), with the reagent A containing BCA and reagent B containing copper sulfate. When mixed, proteins in samples can reduce Cu^2+^ to Cu^+^, resulting in a complex formation with BCA and a color change from green to blue. This color change is proportional to the protein concentration. Then, a standard was prepared at 0.02–2 mg/mL, and a mixture of 25 μL of the standard or sample and 200 μL of the A-B reagent mix was incubated at 37 °C for 30 min for the absorbance measurement at 562 nm for protein quantification.

### 2.10. Measurement of Acetylcholinesterase (AchE) in Rat Cortices

The cortex tissues were homogenized, centrifuged, and then subjected to BCA quantification with the same method described above, and AchE activity was measured using an Elabscience E-E1-0081 kit (Wuhan, China). A total of 50 μL of the standard and sample solution was added to a 96-well plate separately, followed by the addition of 50 μL of Biotinylated detection antibody working solution and incubation at 37 °C for 45 min. After washing three times with washing solution, 100 μL of avidin–horseradish peroxidase (HRP) conjugate working solution was added and incubated at 37 °C for 30 min. The wells were then washed five times, followed by the addition of 90 μL of the substrate reagent and incubation at 37 °C for 15 min, adding 50 μL of stop solution for termination, mixing thoroughly, and the absorbance was measured at 450 nm for quantitation.

### 2.11. Measurement of Aβ40 in the Rat Hippocampus

The hippocampus tissues were homogenized, centrifuged, and then subjected to BCA quantification via the same method described above. Accumulation of Aβ40 protein was measured using a FineTest ER0353 kit (Wuhan, China), adding 100 μL standard and sample into a 96-well plate separately, followed by sealing the plate, incubating for 90 min at 37 °C, washing the plate twice, adding 100 μL of biotin–antibody working solution, sealing the plate, incubating for 60 min at 37 °C, and washing the plate three times, immersing for 1 min each time. Next, 100 μL of HRP–streptavidin conjugate (SABC) working solution was added. The plate was sealed, incubated for 30 min at 37 °C, and washed five times, immersing for 1 min each time. Then, 90 μL of TMB substrate solution was added. The plate was sealed and incubated for 15 min at 37 °C, and 50 μL of stop solution was added for absorbance measurement at 450 nm for calculation.

### 2.12. Measurement of β-Secretase (BACE1) in the Rat Hippocampus

The hippocampus tissues were homogenized and centrifuged and then subjected to BCA quantification with the same method described above. Beta-site amyloid precursor protein cleaving enzyme 1 (BACE1) was measured using a FineTest ER0756 kit (Wuhan, China), adding 100 μL standard and sample into a 96-well plate separately, followed by sealing the plate, incubating for 90 min at 37 °C, washing the plate twice, adding 100 μL of biotin–antibody working solution, sealing the plate, incubating for 60 min at 37 °C, and washing the plate three times, immersing for 1 min each time. Next, 100 μL of SABC working solution was added. The plate was sealed, incubated for 30 min at 37 °C, and washed five times, immersing for 1 min each time. Then, 90 μL of TMB substrate solution was added. The plate was sealed and incubated for 15 min at 37 °C, and 50 μL of stop solution was added for the absorbance measurement at 450 nm for calculation.

### 2.13. Measurement of 8-oxodG in the Rat Hippocampus

The hippocampus tissues were homogenized and centrifuged for measuring the concentration of 8-hydroxy-2′-deoxyguanosine (8-oxodG) using a Cloud-Clone CEA660Ge kit (Houston, TX, USA), adding 50 μL of standard and sample into a 96-well plate separately, followed by adding 50 μL of detection reagent A, sealing the plate, incubating for 60 min at 37 °C, washing the plate three times, adding 100 μL of detection reagent B, sealing the plate, incubating for 30 min at 37 °C, and washing the plate five times, immersing for 1 min each time. Then, 90 μL of TMB substrate solution was added. The plate was sealed and incubated for 15 min at 37 °C, and 50 μL of the stop solution was added for the absorbance measurement at 450 nm for calculation.

### 2.14. Measurement of Antioxidant Enzymes in the Cortices and Livers of Rats

The cortex and liver tissues (0.2 g) were collected separately and washed with PBS buffer (0.01 M, pH 7.4), followed by grinding, homogenizing with 1.8 mL of cell lysis solution on ice, and ultrasonicating for 30 min. Then, the total protein concentration was determined using a BCA kit.

#### 2.14.1. Superoxide Dismutase (SOD)

SOD activity was analyzed with a Cayman 706002 kit (Ann Arbor, MI, USA). Briefly, bovine erythrocyte SOD (Cu/Zn) was used as the standard, and a series of concentrations, including 0.005, 0.010, 0.020, 0.030, 0.040, and 0.050 U/mL, were prepared individually, followed by adding a 10 µL sample and each concentration of the standard to a 96-well plate separately, adding 200 μL of radical detector, mixing well, adding 20 μL of xanthine oxidase to initiate the reaction, shaking for 30 min, and measuring absorbance at 450 nm for the determination of the SOD activity based on the standard curve.

#### 2.14.2. Catalase (CAT)

CAT activity was determined using a Cayman 707002 kit (Ann Arbor, MI, USA). In brief, formaldehyde was used as the standard, and a total of 6 concentrations, including 5, 15, 30, 45, 60, and 75 μM, were prepared individually, followed by adding a 20 μL sample and each concentration of the standard to a 96-well plate separately, adding 30 µL of methanol and 100 µL of buffer, mixing thoroughly, adding hydrogen peroxide (20 µL) to initiate the reaction, shaking for 20 min, adding potassium hydroxide (30 µL) for the reaction termination, adding CAT purpald (30 µL), shaking for 10 min, adding potassium periodate (10 µL) for reaction for 5 min, and measuring the absorbance at 540 nm for the determination of the CAT activity based on the standard curve.

#### 2.14.3. Glutathione Peroxidase (GSH-Px)

GSH-Px activity was determined using a Cayman 703102 kit (Ann Arbor, MI, USA). Briefly, 20 μL of the sample and GSH-Px standard were added to a 96-well plate individually, followed by adding 50 μL of NADPH, 50 μL of buffer, and 50 µL of co-substrate mixture. After mixing thoroughly, 20 μL of cumene hydroperoxide was added to initiate the reaction, followed by shaking for a few seconds, and measuring absorbance at 340 nm once every minute for five minutes. Two measurements were selected to determine the GSH-Px activity of each sample.

### 2.15. Measurement of Dopamine Content in the Brain Striata of Rats

Dopamine content was analyzed with a Fine Biotech EU0392 kit (Wuhan, China). Briefly, we used a wash buffer to wash a 96-well plate coated with the antibody twice, followed by adding, separately, 50 μL of the sample and the standard of each concentration, including 1.562, 3.125, 6.25, 12.5, 25, 50, and 100 ng/mL, adding 50 μL of the biotin-labeled antibody working solution, mixing thoroughly, reacting for 45 min at 37 °C, washing three times with cleaning buffer solution, adding 100 μL of horseradish peroxidase polymer, reacting for 30 min at 37 °C, washing five times with cleaning buffer solution, adding 90 μL of TMB substrate, reacting for 15 min at 37 °C in the dark, adding the stop solution (50 μL), and measuring absorbance at 450 nm for the determination of the dopamine content based on the standard curve.

### 2.16. Measurement of Malondialdehyde (MDA) Content in the Cerebral Cortices and Livers of Rats

The MDA content was analyzed with a Cayman 700870 kit (Ann Arbor, MI, USA). In brief, a series of MDA standard concentrations, including 0.625, 1.25, 2.5, 5, 10, 25, and 50 μM, were prepared individually, followed by collecting, separately, 100 μL of the sample and the standard of each concentration, mixing with 100 μL of the trichloroacetic acid reagent, adding 800 μL of the coloring reagent, heating in a 100 °C water bath for 1 h, cooling for 10 min on ice, centrifuging at 1600× *g* for 10 min (4 °C), collecting the supernatant (200 μL), and adding it to a 96-well plate for an absorbance measurement at 535 nm for the determination of the MDA content based on the standard curve.

### 2.17. Statistical Analysis

All the data were subjected to analysis using Statistical Analysis System (SAS) software (version 6, Cary, NC, USA), involving both analysis of variance (ANOVA) and the Duncan’s multiple range test to determine significance via a mean comparison (*p* < 0.05).

## 3. Results and Discussion

### 3.1. Proximate Analysis of Fresh and Dried Cinnamon Leaves

The proximate analysis of fresh leaves showed carbohydrates to be present in the largest portion (51.53%), followed by moisture (37.24%), crude protein (7.06%), ash (2.86%), and crude fat (1.32%). Comparatively, the carbohydrate content was much higher and the moisture content was lower than that of fresh cinnamon leaves reported in a previous study [14], which could have been caused by the difference in the growth environment in the different locations. However, after drying, the carbohydrate and crude protein contents were higher, which could be attributed to the difference in moisture content. Following the drying of leaves, the carbohydrate content increased to 76.57%, followed by crude protein (10.50%), moisture (6.73%), ash (4.26%), and crude fat (1.96%).

### 3.2. Analysis of CA and the Other Compounds in Cinnamon Leaves and Hydrosol

Following the identification and quantitation criteria described in the Materials and Methods section, a total of 15 compounds, including eugenol, trans-cinnamic acid, coumarin, kaempferol-3-β-D-glucopyranoside, kaempferol, CA, cinnamyl alcohol, quercetin, quercetin-3-O-glucoside, quercetin-3-O-galactoside, rutin, benzoic acid, p-coumaric acid, caffeic acid, and 5-O-caffeoylquinic acid, were identified in cinnamon leaf powder, with retention times from 2.51 min to 11.05 min (Table 1).

As shown in Table 1, all the identified compounds in cinnamon leaf powder were quantified by preparing eight concentrations, including 0.01, 0.02, 0.05, 0.1, 0.2, 0.3, 0.4, and 0.5 µg/g, and analyzed by UPLC-MS/MS. Then, a standard curve was prepared for each functional compound by plotting concentration against peak area, and both the linear regression equation and coefficient of determination (R^2^) were obtained, with the R^2^ of all compounds being >0.9929. The standard curves of all functional compounds and their linear regression equations are provided in the Supplementary Materials. Each functional compound in cinnamon leaf powder and hydrosol was quantified by using the general formula of linear regression equation,

(Y − b)/a × DF,

where a and b refer to the slope and intercept of the linear regression equation, respectively, Y is the peak area of the compounds, and DF is the dilution factor. As the peak of quercetin-3-O-glucoside overlapped with quercetin-3-O-galactoside, the contents of the remaining 14 compounds were higher than the standard curve range (0.01–0.5 µg/g). Consequently, the cinnamon leaf powder extract was diluted to different dilution factors—that is, 10 for compounds 1–4; 100 for compounds 6, 7, 9, 12, and 13; 1000 for compounds 5, 10, 14, and 15; and 100,000 for compound 11. Based on these dilution factors, the contents of 14 compounds provided in Table 1 were diluted to 0.04, 0.24, 0.47, 0.26, 0.07, 0.05, 0.06, 0.21, 0.05, 0.20, 0.07, 0.09, 0.46, and 0.18 µg/g, all of which fall within the standard curve range (0.01–0.5 µg/g) used for quantitation. Similarly, for quantitation in hydrosol, the detected compounds 5, 10, and 14 were diluted 10 times, while compounds 11 and 15, respectively, were diluted 10,000 and 100 times to make the contents of these five compounds in hydrosol 0.42, 0.40, 0.19, 0.12, and 0.15 µg/g, respectively, all of which fall within the standard curve range (0.01–0.5 µg/g).

**Figure 2:**
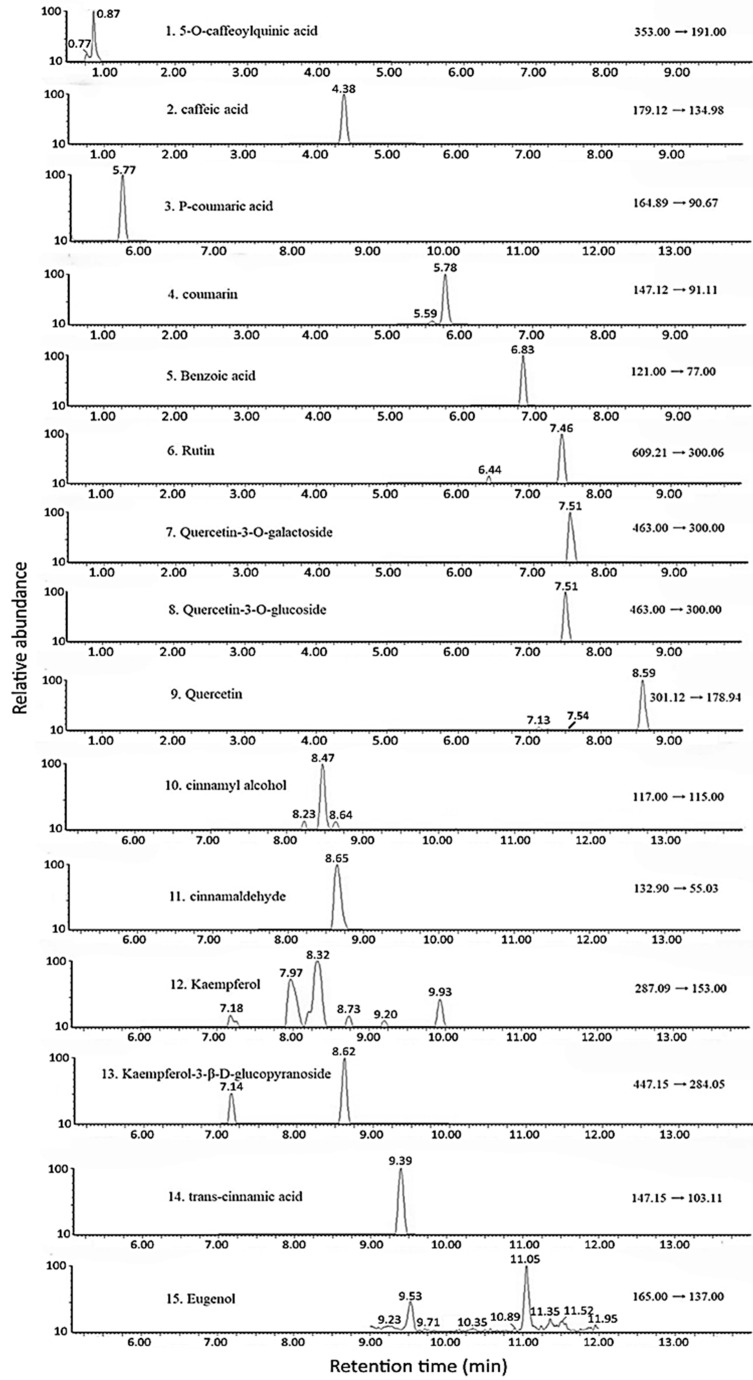
UPLC-MS/MS chromatograms of cinnamaldehyde and the other functional compounds in cinnamon leaf powder as detected by MRM mode.

CA was shown to be present in the highest amount (18,250.7 μg/g), followed by trans-cinnamic acid (464.9 μg/g), eugenol (220.1 μg/g), cinnamyl alcohol (92.2 μg/g), benzoic acid (67.7 μg/g), quercetin (20.9 μg/g), kaempferol-3-β-D-glucopyranoside (19.9 μg/g), kaempferol (4.9 μg/g), p-coumaric acid (4.7 μg/g), coumarin (2.6 μg/g), caffeic acid (2.4 μg/g), and 5-O-caffeoylquinic acid (0.4 μg/g) (Figure 2 and Table 1). But for hydrosol, only five functional compounds were identified, including CA (1218.8 μg/g), eugenol (14.5 μg/g), benzoic acid (4.2 μg/g), cinnamyl alcohol (4.0 µg/g), and trans-cinnamic acid (1.94 µg/g) (Table 1). By comparison, the CA content in cinnamon leaf powder is similar to that reported by Wang et al. [8] and much higher than that reported by Huang and Chen [14], which can be ascribed to differences in the growth location, environmental conditions, and species variety.

It is worth mentioning that this UPLC-MS/MS method was validated, and the method validation data have been reported in our previous publication [14]. Briefly, the recovery of 15 identified compounds ranged from 90.21 to 107.30%, with the RSD ranging from 0.03 to 9.63%, while the RSD value of the intra-day and inter-day variability ranged from 1.93 to 8.21% and from 1.81 to 8.93%, respectively, implying that the quantitation of 15 compounds by the reported method is reliable, as high recovery and precision data were obtained. Moreover, the content of two overlapping peaks of quercetin-3-O-galactoside and quercetin-3-O-glucoside amounted to only 0.03% of the total content of the 15 bioactive compounds (19,161.9 µg/g) presented in Table 1; their overlapping should thus not impact the outcomes/interpretations of this study. Obviously, the dominant bioactive compounds in cinnamon leaves, such as CA, cinnamic acid, and cinnamyl alcohol, are more important in alleviating AD and PD in rats. In addition, their quantitative data is shown as a combination of two compounds in Table 1, and not as a single compound.

### 3.3. Preparation of CLEN and Method Validation

**Figure 3:**
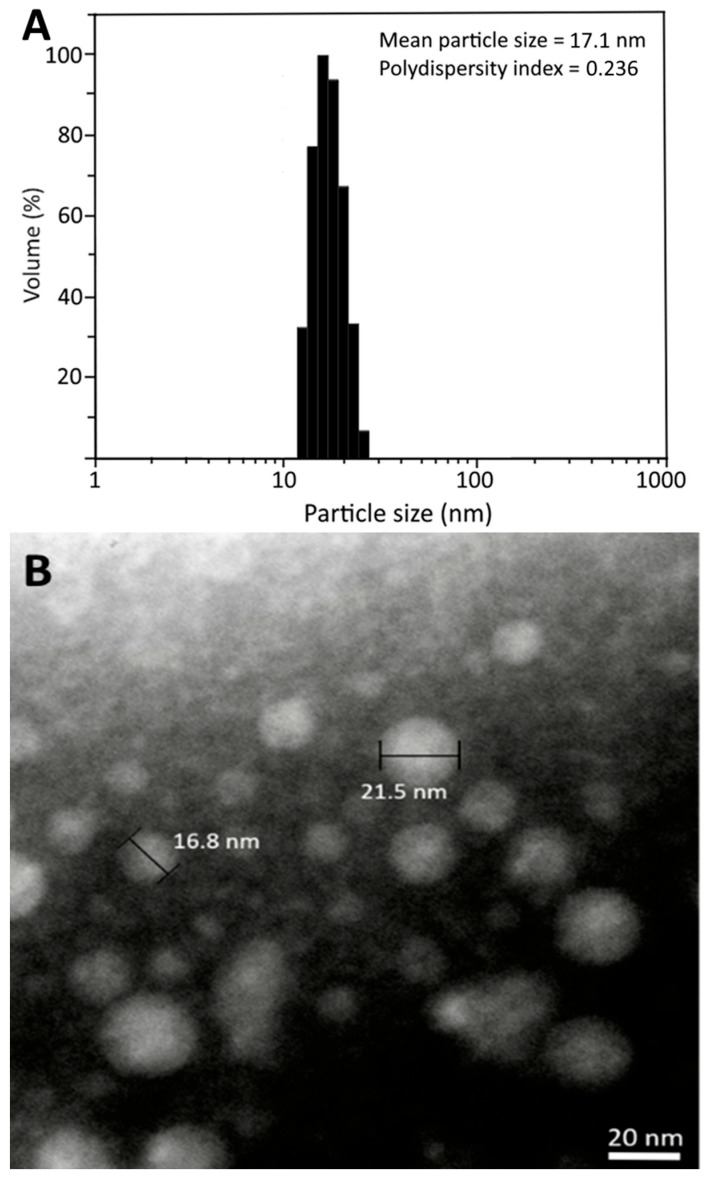
Particle size distribution and shape of CLEN, as determined using a dynamic light scattering (DLS) instrument with an average particle size of 17.1 nm (**A**), along with its TEM image, with an average particle size of 19.2 nm (**B**).

Following the preparation steps described in the Materials and Methods section, the CLEN was successfully prepared, with average particle size and PDI being 17.1 nm and 0.236, respectively, based on DLS analysis (Figure 3A). The TEM analysis also showed a similar average particle size (19.2 nm) with a round shape (Figure 3B). In addition, the zeta potential was −42.68 mV, implying a high stability of CLEN as it conforms to the zeta potential required for high nanoemulsion stability (>30 mV or <−30 mV) [14]. The low PDI (0.236) also indicated a narrow distribution of nanoparticles in CLEN as it was less than 0.3 [8]. Moreover, a high encapsulation efficiency of 90.8% was shown for CA in CLEN, which should be able to prevent the loss of CA during the AD animal experiment.

For method validation in terms of CA encapsulation efficiency, a high recovery of free CA (99.6%) and total CA (104.1%), with RSD at 1.91% and 3.83%, was shown, respectively. The intra-day variability and inter-day variability for free CA was, respectively, 10.29% and 21.00%, with RSD at 3.50% and 4.38%, as well as 21.10% and 21.66%, with RSD at 1.95% and 2.20%, for total CA. Moreover, the encapsulation efficiency was calculated to be 92.9%, which is similar to that (90.8%) before the CA standard was added to the nanoemulsion, demonstrating a high stability of CA in CLEN.

For the reproducibility of CLEN preparation, the RSD values for the particle size, PDI, and zeta potential were 2.48%, 4.55%, and 3.21% for intra-day variability, respectively, while they were 2.42%, 4.68%, and 3.61% for inter-day variability, implying that a high reproducibility was shown for the CLEN preparation reported in this study, with RSD values of all the tested nanoemulsion characteristics being <5%.

### 3.4. Stability of CLEN

Table 2 shows changes in average particle size, PDI, and zeta potential of CLEN during storage at 4 °C for 90 days. Only a slight variation was observed for the average particle size, PDI, and zeta potential over a 90-day storage period at 4 °C, revealing the high stability of this nanoemulsion, which can be attributed to the presence of lecithin and Tween 80, both of which possess high emulsifying power, with the HLB (hydrophile–lipophile balance) of Tween 80 being 15.

### 3.5. Memory Rest for AD Rats Fed with CLE, CLEN, and Powder

**Figure 4:**
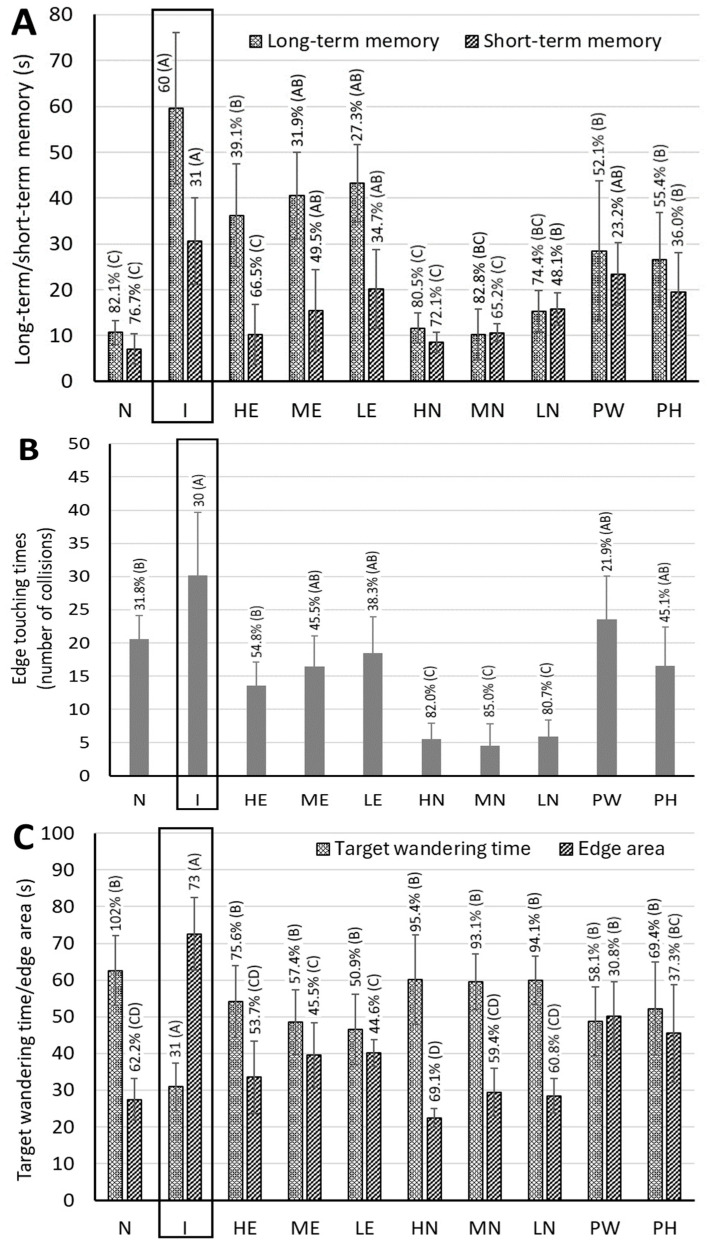

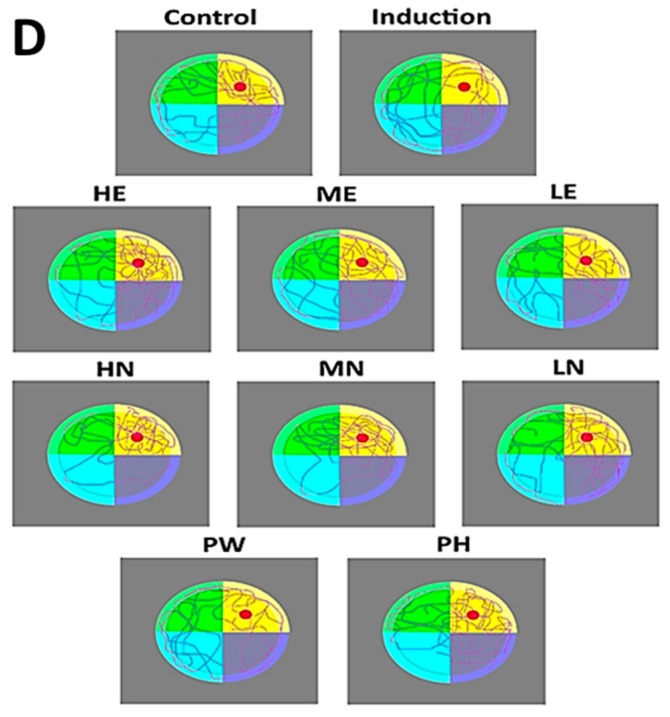
Effects of CLE, CLEN, powder in water (PW), and powder in hydrosol (PH) on long-term and short-term memory (**A**), edge touching times (**B**), target wandering time and edge area (**C**), and the swimming path (**D**) in AD rats. Data are presented as mean ± standard deviation (*n* = 9). N, normal control group; I, induced with Aβ40 solution; HE, high-dose CLE, induced with Aβ40 solution and fed with 90 mg/kg CLE for 4 weeks; ME, medium-dose CLE, induced with Aβ40 solution and fed with 60 mg/kg CLE for 4 weeks; LE, low-dose CLE, induced with Aβ40 solution and fed with 30 mg/kg CLE for 4 weeks; HN, high-dose CLEN, induced with Aβ40 solution and fed with 90 mg/kg CLEN for 4 weeks; MN, medium-dose CLEN, induced with Aβ40 solution and fed with 60 mg/kg CLEN for 4 weeks; LN, low-dose CLEN, induced with Aβ40 solution and fed with 30 mg/kg CLEN for 4 weeks; PW, powder in water, pre-fed with cinnamon leaf powder in water (0.5 g/10 mL) for 4 weeks, followed by induction with Aβ40 solution and feeding with PW (10 mL/kg) for 4 weeks. PH, powder in hydrosol, pre-fed with cinnamon leaf powder in hydrosol for 4 weeks, followed by induction with Aβ40 solution and feeding with PH (10 mL/kg) for 4 weeks. Data with different capital letters (A–D) within each parameter are significantly different at *p* < 0.05.

During the entire animal experiment, we did not observe a significant weight change or behavioral change in rats, implying that the three doses of CLE (30, 60, and 90 mg/kg bw) used in this study were not toxic to rats, and, in particular, that a high dose at 90 mg/kg bw should be safe. Dose selection was based on animal experiments in several published reports and the results of preliminary tests in our lab. Three doses of 30, 60, and 90 mg/kg bw were equivalent to 288, 576, and 864 mg of CA per day, respectively, based on an adult weight with 60 kg. Figure 4A shows the long-term memory and short-term memory test data of AD rats fed with CLE, CLEN, PW, and PH. For the long-term memory test, compared with the control group (N), the induction group had a significantly longer search time (*p* < 0.05) (i.e., by 82.1%), while compared with the induction group, the other groups, including HE, ME, LE, HN, MN, LN, PW, and PH, the search time decreased by 39.1%, 31.9%, 27.3%, 80.5%, 82.8%, 74.4%, 52.1%, and 55.4%, respectively. Apparently all three CLEN treatments showed better long-term memory than the other CLE and powder treatments (PW and PH), with no significant difference (*p* > 0.05) observed between the control and CLEN treatments. A similar outcome was observed for the short-term memory test data, with both high-dose and medium-dose CLEN showing the most pronounced effect in improving short-term memory and being comparable to the control treatment (Figure 4A). In addition, there was no significant difference (*p* > 0.05) between the control and high-dose CLE treatments, which also showed better short-term memory than the other CLE (ME and LE) and powder (PW and PH) treatments.

Figure 4 also shows the spatial probe test data of AD rats fed with CLE, CLEN, PW, and PH. Compared with the control group, the induction group had significantly more collisions (*p* < 0.05), with longer edge touching time, while compared with the induction group, the other treatments, including HE, ME, LE, HN, MN, LN, PW, and PH, showed a declined collision by 54.8%, 45.5%, 38.3%, 82.0%, 85.0%, 80.7%, 21.9%, and 45.1%, respectively (Figure 4B). By comparison, all three CLEN treatments showed a much shorter edge touching time than the other CLE (HE, ME, LE) and powder (PW, PH) treatments, implying that rats receiving CLEN could find a resting platform more readily with minimal collisions. A similar outcome was observed for the target quadrant wandering time, as evident by increases of 102%, 75.6%, 57.4%, 50.9%, 95.4%, 93.1%, 94.1%, 58.1%, and 69.4% for the control, HE, ME, LE, HN, MN, LN, PW, and PH treatments, respectively (Figure 4C). This result accords with the edge touching time, with rats receiving CLEN treatments spending less time in the edge area. Likewise, for the edge area, significant reductions of 62.2%, 53.7%, 45.5%, 44.6%, 69.1%, 59.4%, 60.8%, 30.8%, and 37.3% were shown for the control, HE, ME, LE, HN, MN, LN, PW, and PH treatments, respectively (Figure 4C). Similarly to the result of the target quadrant wandering time, rats with CLEN treatments spent more time in the target area, revealing their ability to find the target areas within a short period of time. This finding is in line with the swimming paths of rats fed with CLE, CLEN, PW, and PH (Figure 4D), with the CLEN treatment showing more dense paths in the target area compared to CLE and powder treatment.

It is worth pointing out that both PW and PH were effective in improving long-term memory and short-term memory, with a shorter edge touching time, longer target quadrant wandering time, and less time spent in the edge area. Comparatively, PH showed a more pronounced effect in improving rat memory than PW, which can be attributed to the presence of a higher level of CA in PH (18,250.7 µg/g in powder and 1218.8 µg/g in hydrosol). This finding further demonstrated that the pre-feeding of rats with PW or PH for 4 weeks was efficient in possibly preventing AD occurrence.

In several previous reports, Do et al. [25] studied the effect of trans-cinnamaldehyde (TCA) on improving cognitive impairment in 5X FAD mice, with both Morris water maze and passive avoidance tests being used for evaluation. A time reduction in finding the escape platform was shown for the former, and the entry into the dark room was reduced for the latter. Similarly, the galactose- and AlCl_3_-induced cognitive dysfunction in mice as affected by TCA was studied by Ryu et al. [26], reporting that the time in finding the escape platform was reduced based on the Morris water maze test, while the difference in the swimming speed and total swimming distance between each group was not significant (*p* > 0.05). Another study also proved that the administration of the H_2_O-soluble cinnamon extract was effective in protecting the neurotransmission system in AlCl_3_-induced rats [27]. Apparently, both TCA and H_2_O-soluble cinnamon extract should be effective in improving cognitive function in AD rats or mice.

### 3.6. Measurement of Biological Activity Indicators in the Hippocampi of Rat Brains

**Figure 5:**
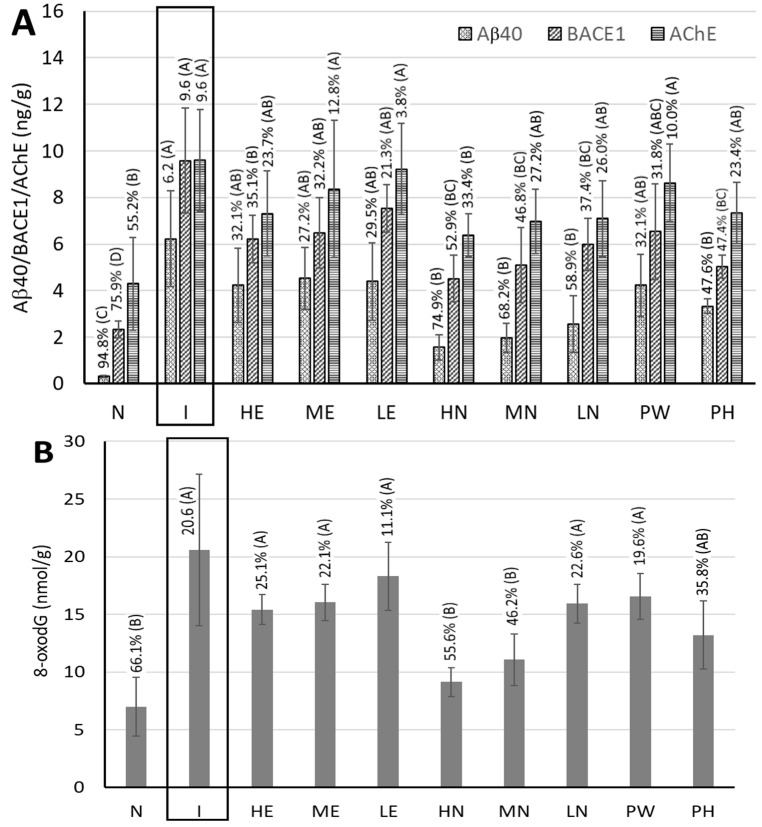
Effects of CLE, CLEN, powder in water (PW), and powder in hydrosol (PH) on the contents of Aβ40 and BACE1 in the rat hippocampus and AChE in the rat cerebral cortex (**A**), as well as 8-oxodG in the rat hippocampus (**B**). Data are presented as mean ± standard deviation (*n* = 9). N, normal control group; I, induced with Aβ40 solution; HE, high-dose CLE, induced with Aβ40 solution and fed with 90 mg/kg CLE for 4 weeks; ME, medium-dose CLE, induced with Aβ40 solution and fed with 60 mg/kg CLE for 4 weeks; LE, low-dose CLE, induced with Aβ40 solution and fed with 30 mg/kg CLE for 4 weeks; HN, high-dose CLEN, induced with Aβ40 solution and fed with 90 mg/kg CLEN for 4 weeks; MN, medium-dose CLEN, induced with Aβ40 solution and fed with 60 mg/kg CLEN for 4 weeks; LN, low-dose CLEN, induced with Aβ40 solution and fed with 30 mg/kg CLEN for 4 weeks; PW, powder in water, pre-fed with cinnamon leaf powder in water (0.5 g/10 mL) for 4 weeks, followed by induction with Aβ40 solution and feeding with PW (10 mL/kg) for 4 weeks. PH, powder in hydrosol, pre-fed with cinnamon leaf powder in hydrosol for 4 weeks, followed by induction with Aβ40 solution and feeding with PH (10 mL/kg) for 4 weeks. Data with different capital letters (A–D) within each parameter are significantly different at *p* < 0.05.

Figure 5A shows the amount of Aβ40 deposition in the hippocampi of rat brains. Compared with the control group, the induction group demonstrated a significantly higher amount of Aβ40 deposition (*p* < 0.05) by 94.8%, while compared with the induction group, the other groups including, HE, ME, LE, HN, MN, LN, PW, and PH, showed a reduction in Aβ40 by 32.1%, 27.2%, 29.5%, 74,9%, 68.2%, 58.9%, 32.1%, and 47.6%, respectively. Obviously, the CLEN treatments were more effective than CLE and powder treatments in decreasing Aβ40 deposition, especially for the high-dose CLEN. This outcome is similar to those of several previous studies showing that both TCA and cinnamon water extract could reduce Aβ40 deposition in mice [25] and rats [27].

Figure 5A also shows the BACEl contents in the hippocampi of rat brains. Compared with the control group, the induction group had a significantly higher BACEl content (*p* < 0.05) (i.e., by 75.9%), while compared with the induction group, the other groups, including HE, ME, LE, HN, MN, LN, PW, and PH, showed a decline in BACE1 by 35.1%, 32.2%, 21.3%, 52.9%, 46.8%, 37.4%, 31.8%, and 47.4%, respectively. Comparatively, the high-dose CLEN showed a more distinct effect in inhibiting BACE1 activity than the other CLE, CLEN, and powder treatments. As BACEl is a vital enzyme in decomposing the Aβ40 protein precursor for the generation of Aβ40, the inhibition of BACE1 can be expected to reduce Aβ40 deposition.

The effects of CLE, CLEN, PW, and PH on 8-oxodG content, a DNA oxidation product for the evaluation of mitochondrial function, in the hippocampi of rat brains are shown in Figure 5B. Compared with the control group, the induction group had a significantly higher 8-oxodG content (*p* < 0.05) (i.e., by 66.1%), while compared with the induction group, the other groups, including HE, ME, LE, HN, MN, LN, PW, and PH, showed decreases of 25.1%, 22.1%, 11.1%, 55.6%, 46.2%, 22.6%, 19.6%, and 35.8%, respectively. Obviously both high-dose and medium-dose CLEN showed a more significant effect in inhibiting 8-oxodG formation than the other CLE and powder treatments, thereby improving mitochondrial function.

By comparison, PH was more effective than PW in reducing Aβ40 deposition and the contents of BACE1 and 8-oxodG in the hippocampi of rat brains, which should mainly be caused by the presence of the higher level of CA in PH, as mentioned above. More importantly, this outcome provides significant evidence that the intake of PH or PW should be efficient in possibly preventing AD occurrence.

### 3.7. Measurement of Acetylcholinesterase (AchE) Activity in the Cerebral Cortices of Rat Brains

Figure 5A shows the effect of CLE, CLEN, PW, and PH treatments on AchE activity in the cerebral cortices of rat brains. Acetylcholine is a vital neurotransmitter in brain, and AchE inhibitors are often used as drugs for the early treatment of patients with AD. Compared with the control group, the induction group had a significantly higher AchE content (i.e., by 55.2%), while compared with the induction group, reductions of 23.7%, 12.8%, 3.8%, 33.4%, 27.2%, 26.0%, 10.0%, and 23.4% were found for HE, ME, LE, HN, MN, LN, PW, and PH, respectively. Comparatively, the CLEN treatments were more efficient in decreasing AchE activity than the other CLE and powder treatments, especially for the high-dose CLEN. In addition, both PH and PW were effective in inhibiting AchE activity, with the former showing a more distinct effect, revealing the AD-retarding potential of consumption of PH and PW.

### 3.8. Measurement of Activities of Antioxidant Enzymes in the Cerebral Cortices of Rat Brains

As the brain contains about 60% fat and consumes a large amount of adenosine triphosphate (ATP) to maintain physical activity during work, its demand for oxygen is high, so it produces a large quantity of ROS; this causes oxidative stress, peroxidation of unsaturated fatty acids in fats, mitochondrial dysfunction, the loss of brain cell membrane integrity, and brain cell apoptosis [28]. Furthermore, the excessive production of ROS has been proven to be the main cause of neurodegenerative diseases such as PD and AD [2]. Thus, it is imperative to determine antioxidant enzyme activities in the cortices of rat brains to elaborate their roles in the progress of AD.

**Figure 6:**
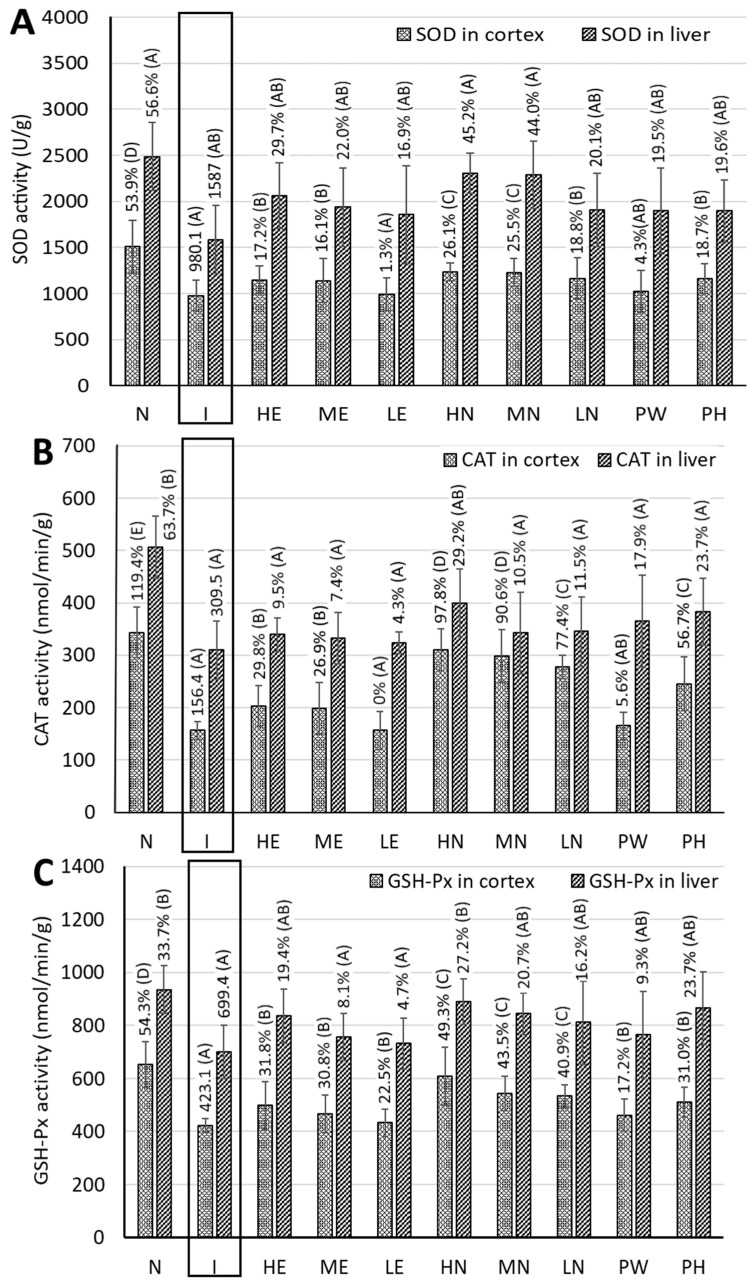

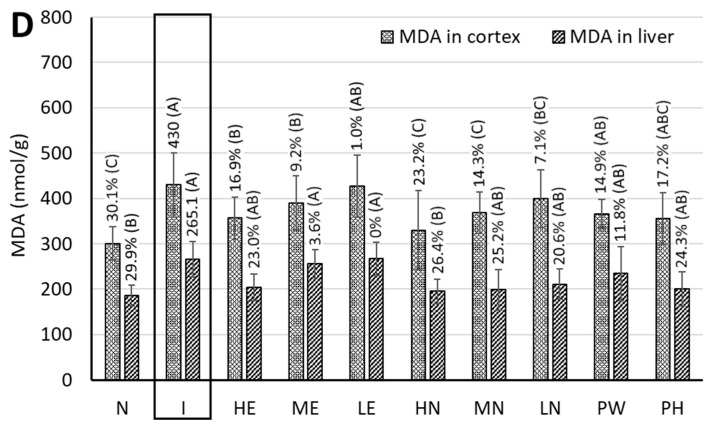
Effects of CLE, CLEN, powder in water (PW), and powder in hydrosol (PH) on the contents of SOD (**A**), CAT (**B**), GSH-Px (**C**), and MDA (**D**) in the brain cortices and livers of rats. Data are presented as mean ± standard deviation (*n* = 9). N, normal control group; I, induced with Aβ40 solution; HE, high-dose CLE, induced with Aβ40 solution and fed with 90 mg/kg CLE for 4 weeks; ME, medium-dose CLE, induced with Aβ40 solution and fed with 60 mg/kg CLE for 4 weeks; LE, low-dose CLE, induced with Aβ40 solution and fed with 30 mg/kg CLE for 4 weeks; HN, high-dose CLEN, induced with Aβ40 solution and fed with 90 mg/kg CLEN for 4 weeks; MN, medium-dose CLEN, induced with Aβ40 solution and fed with 60 mg/kg CLEN for 4 weeks; LN, low-dose CLEN, induced with Aβ40 solution and fed with 30 mg/kg CLEN for 4 weeks; PW, powder in water, pre-fed with cinnamon leaf powder in water (0.5 g/10 mL) for 4 weeks, followed by induction with Aβ40 solution and feeding with PW (10 mL/kg) for 4 weeks. PH, powder in hydrosol, pre-fed with cinnamon leaf powder in hydrosol for 4 weeks, followed by induction with Aβ40 solution and feeding with PH (10 mL/kg) for 4 weeks. Data with different capital letters (A–E) within each parameter are significantly different at *p* < 0.05.

Figure 6A shows the SOD activity in cortices of rat brains. Compared with the control group, the induction group had a significantly lower SOD activity (i.e., by 53.9%), while compared with the induction group, the other groups, including, HE, ME, LE, HN, MN, LN, PW, and PH, showed increments of 17.2%, 16.1%, 1.3%, 26.1%, 25.5%, 18.8%, 4.3%, and 18.7%, respectively. This outcome implied that the CLEN treatments showed a higher SOD activity than the other CLE and powder treatments. A similar result was reported by Ryu et al. [26], studying the effect of TCA on cognitive improvement in AD mice induced by d-galactose and AlCl_3_ following a treadmill test. In eukaryotes, SOD can combine with copper and zinc ions to convert superoxide free radicals into the less toxic hydrogen peroxide and oxygen.

Figure 6B shows the CAT activity, catalyzing the decomposition of hydrogen peroxide into water and oxygen, in the cortices of rat brains. Compared with the induction group, the other groups, including control, HE, ME, HN, MN, LN, PW, and PH, showed a much higher CAT activity by 119.4%, 29.8%, 26.9%, 97.8%, 90.6%, 77.4%, 5.6%, and 56.7%, respectively. This result revealed that the CLEN treatments possessed higher CAT activity than the other CLE and powder treatments, especially for the high-dose CLEN. However, the differences in CAT activity between the induction group and LE and PW were not significant (*p* > 0.05). A similar trend was shown for the GSH-Px activity in the cortices of rat brains (Figure 6C), with higher activity by 54.3%, 31.8%, 30.8%, 22.5%, 49.3%, 43.5%, 40.9%, 17.2%, and 31.0% being shown for control, HE, ME, LE, HN, MN, LN, PW, and PH, respectively. Similarly to SOD and CAT activities, the CLEN treatments exhibited higher GSH-Px activity than the other CLE and powder treatments, especially for the high-dose CLEN, which should be the most efficient in minimizing the oxidative stress caused by the accumulation of ROS in brain tissue. Comparatively, PH showed a more significant effect than PW in elevating the activities of SOD, CAT, and GSH-Px in the cortices of rat brains, indicating the ability of PH to reduce oxidative stress in brain and thereby having a possible preventive effect on AD.

### 3.9. MDA Content in the Cortices of Rat Brains

MDA is a major degradation product from lipid peroxides during oxidation and can serve as a measure of the degree of lipid oxidation and oxidative stress. The MDA contents in the cortices of rat brains are shown in Figure 6D, with decreases of 30.1%, 16.9%, 9.2% 1.0%, 23.2%, 14.3%, 7.1%, 14.9%, and 17.2% being shown for control, HE, ME, LE, HN, MN, LN, PW, and PH, respectively. By comparison, only the high-dose CLEN showed a lower MDA content than the other extract and powder treatments, indicating the great potential of this nanoemulsion in attenuating oxidative stress for possible AD treatment. In addition, like the antioxidant enzyme activity, PH was more effective than PW in reducing oxidative stress, as evidenced by the lower level of MDA in the cortices of rat brains.

### 3.10. Dopamine Contents in the Brain Striata of Rats

**Figure 7:**
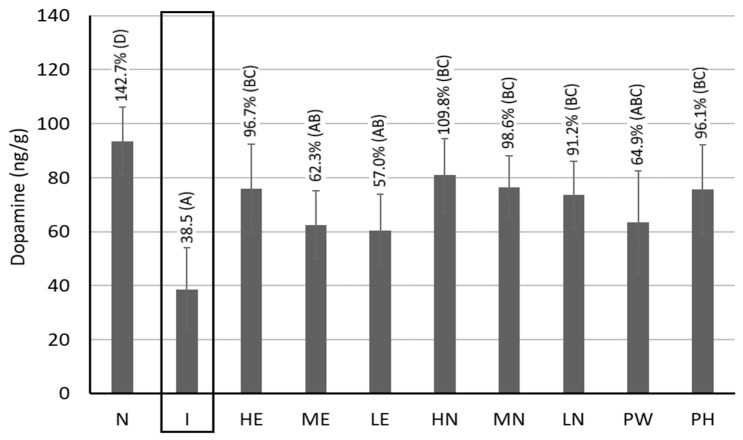
Effects of CLE, CLEN, powder in water, and powder in hydrosol on the content of dopamine in the rat brain striatum. Data are presented as mean ± standard deviation (*n* = 9). N, normal control group; I, induced with Aβ40 solution; HE, high-dose CLE, induced with Aβ40 solution and fed with 90 mg/kg CLE for 4 weeks; ME, medium-dose CLE, induced with Aβ40 solution and fed with 60 mg/kg CLE for 4 weeks; LE, low-dose CLE, induced with Aβ40 solution and fed with 30 mg/kg CLE for 4 weeks; HN, high-dose CLEN, induced with Aβ40 solution and fed with 90 mg/kg CLEN for 4 weeks; MN, medium-dose CLEN, induced with Aβ40 solution and fed with 60 mg/kg CLEN for 4 weeks; LN, low-dose CLEN, induced with Aβ40 solution and fed with 30 mg/kg CLEN for 4 weeks; PW, powder in water, pre-fed with cinnamon leaf powder in water (0.5 g/10 mL) for 4 weeks, followed by induction with Aβ40 solution and feeding with PW (10 mL/kg) for 4 weeks. PH, powder in hydrosol, pre-fed with cinnamon leaf powder in hydrosol for 4 weeks, followed by induction with Aβ40 solution and feeding with PH (10 mL/kg) for 4 weeks. Data with different capital letters (A–D) are significantly different at *p* < 0.05.

A decrease in the dopamine level in brain is a vital index of PD caused by the malfunction of brain cells and the accumulation of α-synuclein, resulting in severe oxidative stress, cell membrane damage, and neuron death. Figure 7 shows the dopamine contents in the brain striatum of rats. Compared with the induction group, a much higher content of dopamine by 142.7%, 96.7%, 62.3%, 57.0%, 109.8%, 98.6%, 91.2%, 64.9%, and 96.1% was shown for control, HE, ME, LE, HN, MN, LN, PW, and PH, respectively. Comparatively, both high-dose and medium-dose CLEN showed the most distinct effect in elevating the dopamine contents, thereby possessing notable potential in treating patients with PD. This result agrees with that of a previous study by Wang et al. [8], demonstrating that the CLEN was effective in alleviating PD in rats through the substantial elevation of the dopamine contents. More importantly, a much lower level of dopamine was observed in Aβ40-induced rats, revealing that the AD occurrence can be accompanied by PD development. Furthermore, PH showed a more prominent effect than PW in raising dopamine level; thus, it possesses notable potential in possibly preventing PD occurrence. The presence of a higher level of CA in PH could be responsible for this effect.

### 3.11. Measurement of Antioxidant Enzyme Activities and MDA Content in Rat Livers

Like the brain, the liver is an important organ for body detoxification, and the excessive production of ROS in the liver can cause oxidative stress accumulation, which, in turn, can impair brain function. Moreover, the liver was found to be the primary peripheral organ involved in β-amyloid metabolism, playing a vital role in the pathophysiology of AD as the impaired cholesterol metabolism in the liver may exacerbate AD development [29]. Therefore, the antioxidant enzyme activities in rat livers need to be measured. Figure 6A shows the SOD activity in rat livers, with increases of 56.6%, 29.7%, 22.0%, 16.9%, 45.2%, 44.0%, 20.1%, 19.5%, and 19.6% being shown for control, HE, ME, LE, HN, MN, LN, PW, and PH, respectively, compared to the induction group. Obviously, both high-dose and medium-dose CLEN showed a much higher SOD activity than the other CLE and powder treatments. Likewise, higher CAT activities, i.e., by 63.7%, 9.5%, 7.4%, 4.3%, 29.2%, 10.5%, 11.5%, 17.9%, and 23.7%, were shown for control, HE, ME, LE, HN, MN, LN, PW, and PH, respectively (Figure 6B). Comparatively, only the high-dose CLEN showed a higher CAT activity than the other CLE and powder treatments. A similar tendency was observed for GSH-Px activity, with increments of 33.7%, 19.4%, 8.1%, 4.7%, 27.2%, 20.7%, 16.2%, 9.3%, and 23.7% being shown for control, HE, ME, LE, HN, MN, LN, PW, and PH, respectively (Figure 6C). In comparison with both CLE and powder treatments, the high-dose CLEN showed the most pronounced effect in raising the GSH-Px activity and decreasing the MDA content, while there is no significant difference (*p* > 0.05) in the MDA content between the control and the HE, HN, MN, LN, PW, or PH groups (Figure 6D). This finding further demonstrates that the administration of rats with CLE, CLEN, PH, or PW could elevate the liver function and thereby improve the brain function of rats. Similarly to the result of the antioxidant enzyme activity in the rat brain cortex, PH showed a higher level of SOD, CAT, and GSH-Px than PW and should be more efficient in reducing oxidative stress in the liver for possible AD prevention.

As mentioned above, CA is the dominant functional compound in cinnamon leaves. But because of the poor water solubility of CA, its stability and bioavailability may be reduced in vivo. Therefore, we prepared CLEN for CA encapsulation in this study, aiming to enhance its in vivo bioactivity for AD improvement in rats. In addition to CLEN, the pre-intake of PH or PW for 4 weeks was found to be effective in ameliorating AD and PD in rats, demonstrating the possible preventive effects of PH and PW against both diseases, with the former being superior to the latter. In several published reports employing cinnamon oil as a raw material for nanoemulsion preparation, average particle sizes of 176 nm, 65 nm, and 96.39 nm have been obtained by Davila-Rodriguez et al. [30], Ghosh et al. [31], and Mukerjee et al. [32], respectively. Comparatively, the average particle size of CLEN in our study is much smaller, which can be attributed to the presence of lecithin and Tween 80 in an appropriate proportion, as both are frequently used surfactants that can decrease the average particle size of the nanoemulsion when used in combination. In addition, similarly to two reports published by Huang and Chen [14] and Wang et al. [8], with CLE employed as raw material for nanoemulsion preparation, a high storage stability for 90 days at 4 °C was obtained.

As indicated before, this study is a continuation of our prior work [8,14] dealing with the preparation of a similar nanoemulsion system with an average particle size of 36.58 nm and 30.1 nm, showing substantial improvements in rats with type II diabetes and PD, respectively. By comparison, in this study, the average particle size (17 nm) was much smaller, which should be able to cross the BBB more effectively to elevate brain cell viability and thereby lead to significant improvements in rats with AD. The correlation between type II diabetes and PD or AD has been documented, with the main common factors being oxidative stress, insulin resistance, the formation of advanced glycation end products, inflammation, mitochondria dysfunction, and autophagic impairment [17,18,21]. For instance, Olorunnado et al. [33] reported that trans-cinnamaldehyde could attenuate the diabetes-induced impairment of learning and memory in a female Wistar rat model with insulin resistance through a reduction in the levels of TNF-α and NF-κB in the rat hippocampus based on Morris water maze and Y-maze tests. Similarly, the two main compounds in essential oil from *Cinnamomum zeylanicum*, TCA and cinnamyl acetate, have been shown to alleviate neurodegenerative symptoms such as pyknosis and astrogliosis in the rat hippocampus through the inhibition of monoamine oxidase and cholinesterase, thereby reducing Aβ40 aggregation in the rat brain [34]. Concerning the relationship between type II diabetes and PD, mitochondrial dysfunction has been shown in insulin-resistant mice, resulting in an increase in α-synuclein level and a decrease in dopamine level [35,36]. Thus, the nanoemulsion system developed in our study possesses great potential for the possible treatment of PD and type II diabetes.

As for the association between PD and AD, there are several similarities and differences based on published reports. Both can cause depression, sleep disturbance, and anxiety in the early stages, while in the later stages, both diseases may lead to hallucination, delusion, and other psychotic symptoms. Additionally, the dementia symptoms of AD will not subside, while the PD dementia symptoms can come and go day by day. Specifically, as stated above, PD is closely related to the basal ganglia degeneration and buildup of α-synuclein in the brain, along with hippocampus and thalamus degeneration, while AD is associated with a buildup of neurofibrillary tangles and Aβ plaques in the brain, concomitant with hippocampus degeneration [37]. As patients with PD may possess the AD-like symptom of brain atrophy, Charisse et al. [37] conducted a clinical trial exploring the association between PD and AD, with 178 PD patients and 84 healthy subjects enrolled in the study. The results showed that the MRI indices of brain aging and AD-like brain atrophy are differentially associated with domain-specific cognitive performance and motor disease severity in PD, implying that the elevated expression of the AD-like symptom of brain atrophy can be a key imaging feature of cognitive decline in PD, with both PD and AD possibly contributing to cognitive impairment by affecting the working memory and attention domain. However, as no cerebrospinal fluid and histopathological biomarkers were measured in this study, a conclusion can be difficult to reach regarding the possible underlying pathomechanisms based on the imaging findings.

Among the various biomarkers used for evaluation of AD and PD, there are many similarities. For example, the pro-oxidants iron and aluminum, as well as the lipid oxidation product, MDA, responsible for oxidative stress, were shown to increase substantially in the brains of patients with AD and PD, with the main accumulation sites being neurofibrillary tangles in AD and Lewy bodies in PD [21]. But for the antioxidant enzyme activity, GSH-Px, CAT, and SOD showed a significant increase or insignificant change depending on the brain regions in both AD and PD patients, probably caused by a compensatory response to oxidative stress, with the oxidative changes being the most prominent in the medial temporal lobe, where histopathologic alterations are most severe [21,38]. This finding suggests that antioxidant enzyme activity may be an insignificant biomarker in evaluating the progress of AD and PD. Nevertheless, α-synuclein accumulation did occur in PD and AD, probably caused by a change in the folding pattern of α-synuclein from α-helix to β-sheet, resulting in a pathogenic aggregation of the mutant protein [21]. Additionally, a marker for nitric oxide formation, 3-nitrotyrosine, was found to increase in the central core of the Lewy bodies in PD patients and in the neurofibrillary tangles in AD patients, while a decline in the mitochondrial membrane potential was observed in fibroblasts derived from PD patients and in stem cells transfected to overexpress amyloid precursor protein in familial AD [21]. This finding further demonstrates that both oxidative stress and mitochondrial dysfunction play a key role in the pathogenesis of AD and PD. In our study, a substantial reduction in MDA and 8-oxodG contents was observed following administration of CLEN, revealing the great potential of this nanoemulsion in treating AD and PD patients.

The CLEN prepared in our study is composed of 1% soybean oil, 2% lecithin, 6% Tween 80, 91% deionized water, and CLE. Soybean oil is rich in linoleic acid (49–53%) and linolenic acid (5–9%), both of which are essential fatty acids reported to play a vital role in maintaining the functions of nervous and immune systems and inflammation, as well as the integrity of the brain cell membrane and skin health [39]. More recently, the associations of plasma ω-3 fatty acid (eicosapentaenoic acid (EPA), docosahexaenoic acid (DHA), linolenic acid), and ω-6 fatty acid (linoleic acid) with the incidence of cancers in a large prospective cohort with 253,138 UK Biobank participants were explored by Zhang et al. [40], demonstrating that participants with higher levels of ω-3 fatty acid had lower rates of colon, stomach, lung, and digestive tract cancers, while those with high levels of ω-6 fatty acid had lower rates of brain, malignant melanoma, and bladder cancers. Lecithin, a natural antioxidant and surfactant widely used for functional food production, is rich in phosphatidylcholine, which can assist in repairing the cell membrane and releasing choline in the intestine for subsequent synthesis of acetylcholine in the brain, a vital neurotransmitter in maintaining brain cell health [41]. It has been reported that deficiency in the acetylcholine level could result in an increase in butyrylcholinesterase (BuChE) content, a vital enzyme in the pathogenesis of AD, leading to an unbalanced AchE–BuChE ratio [9]. Tween 80, a frequently used surfactant with high HLB, can be used to produce a nanoemulsion with small particle size, narrow particle size distribution, and high stability [42]. More importantly, Tween 80 has been shown to be effective in inhibiting the growth of various types of cancer cells and facilitating the adsorption of apolipoprotein E (ApoE) from the blood to the brain for the subsequent conjugation of ApoE, with a low-density lipoprotein (LDL) receptor on the brain cell membrane employed to speed up the crossing of the BBB for the activation of brain cells [8,43].

In addition to soybean oil, lecithin, and Tween 80, the dominant functional compound in cinnamon leaves, CA, also plays an important role in alleviating AD in rats. In several previous studies, Tepe and Ozasalan [34] reported that CA was effective in alleviating AD in rats through a reduction in the self- and Cu^2+^-induced accumulation of Aβ1-42 by 57.78% and 84.53%, respectively, as well as the inhibition of monoamine oxidase (MAO) A and B by 96.32% and 96.29%, respectively. In another study, the improvement of spatial memory deficits and anxiety-like behavior in intracerebroventricular streptozotocin (STZ)-induced rats through a decline of hippocampal Aβ accumulation was found for CA at 100 mg/kg by modulating the pathway of hippocampal insulin-resistant substrate-1/serine or threonine-specific protein kinase/glycogen synthase kinase-3 beta (IRS-I/Akt/GSK-3β) phosphorylation [44]. GSK-3β has been demonstrated to be responsible for AD disorders caused by the deposition of Aβ plaques [42]. In an STZ-induced AD rat model, the behavior performance was improved for the Morris water maze test following the administration of the CA extract and insulin injection, which can be attributed to the synergistic effect of the combination of the CA extract and insulin through the regulation of dyslipidemia and the insulin signaling pathway for the elevation of expressions of GLUT1, -3, and -4 genes in the hippocampal tissue [45].

Another important factor affecting Aβ generation is BACE1 (β-secretase) activity, which can degrade Aβ precursor protein. In a study dealing with the effect of TCA on Aβ aggregation in 5XFAD mice, TCA was shown to downregulate the β-secretase expression, accompanied by an increase in the expressions of the β-secretase regulators peroxisome proliferator-activated receptor r, coactivator 1α, and silent information regulator 1 for subsequent reduction in Aβ deposition, revealing the potential of TCA as a therapeutic drug in AD treatment [18]. Similarly, CA treatment was shown to improve memory impairment in mice induced by cholinergic blockade through the inhibition of hippocampal Akt and MAPK dysregulations [46].

Based on the published reports, most CA was converted to cinnamic acid in the liver following intake, which, in turn, produced sodium benzoate through β-oxidation for entrance into the BBB to inhibit brain cell inflammation [47]. Additionally, sodium benzoate exerted the neurotrophic effect through activation of both protein kinase A (PKA) and cyclic adenosine monophosphate (cAMP) response element binding protein in vivo in the central nervous system. Furthermore, cinnamon and its metabolite sodium benzoate was found to improve neurodegeneration through the elevation of the levels of brain-derived neurotrophic factor (BDNF) and neurotrophin-3 (NT-3) dose-dependently in human neurons and astrocytes, as well as through the protection of protein deglycase DJ-1, also known as Parkinson disease protein 7, and parkin, a 465-amino acid residue E3 ubiquitin ligase, which plays a critical role in ubiquitination—the process whereby molecules are covalently labeled with ubiquitin and directed towards degradation in proteasomes or lysosomes [47,48].

As pointed out above, the primary factor responsible for the neuronal apoptosis of brain cells can be oxidative stress. Specifically, oxidative stress can occur via excessive accumulation of reactive oxygen species (ROS), which can be generated during mitochondrial electron transport, cell proliferation, the accumulation of metal ions (especially iron), the enzymatic catalysis of phagocytes, and cell autophagy [2]. Moreover, the brain has been shown to be susceptible to oxidative damage due to the presence of high contents of fat and polyunsaturated fatty acid, especially arachidonic acid and DHA, as well as low levels of antioxidant enzymes [49]. Thus, providing AD and PD patients with an optimal variety and adequate quantity of antioxidants can be another approach to treating AD and PD in the early stages. Many studies have shown that the major functional compounds in cinnamon, such as CA, TCA, cinnamic acid, and eugenol, could act as antioxidants to inhibit the brain’s oxidative stress and restore autophagy function, thereby retarding the progress of AD and PD [4,50].

The BBB, composed of brain microvascular endothelial cells, pericytes, and astrocytes, is a physical and biological barrier between the central nervous system and peripheral circulation, with tight junctions forming a highly specialized intercellular adhesion complex in endothelial and epithelial cells [51]. Significantly, the impairment of tight junction integrity caused by the abnormal expression of tight junction-related proteins leads to an increase in BBB permeability, which is closely associated with brain disease such as AD as BBB dysfunction has been shown to promote Aβ40 production through the activation of BACE1 and γ-secretase [51]. Thus, it is essential to maintain the normal transport function of the BBB. The passage of molecules through the tight junction, composed of lipid bilayers and a complex network of proteins, can be based on lipophilicity and size, with molecules with molecular weights (MWs) of less than 400 Da (g/mol) being shown to readily cross the BBB via paracellular diffusion [52]. Interestingly, the major functional compounds in the CLEN include CA, eugenol, cinnamic acid, cinnamyl alcohol, and benzoic acid, all of which are lipophilic, with MW < 400 Da, resulting in a more efficient crossing because of the BBB’s lipid membrane nature. Additionally, the hydrophilic Tween 80 used for the preparation of the nanoemulsion can cross the BBB through receptor-mediated transport, as mentioned above. Similarly, lecithin can assist in facilitating the crossing of the BBB through binding with APoE for subsequent conjugation with receptors on the brain cell membrane, followed by uptake by endothelial cells via transcytosis while bypassing the lysosomal degradation pathways and delivering the functional compound to the brain [53]. But for linoleic acid and linolenic acid, both are long-chain fatty acids, often bound to albumin in the bloodstream, which can also cross the BBB via passive diffusion through detachment from albumin or via protein-mediated transport through membrane proteins. Regarding the optimal size of a nanosystem to cross the BBB, Betzer et al. [54] reported that a nanogold size of 20 nm was the most effective in the crossing the BBB, followed by sizes of 50 nm and 70 nm. However, in another study dealing with the delivery of nanoparticles to brain, a size of 15 nm was shown to be the most efficient in crossing the BBB, followed by 3 nm and 120 nm, probably caused by the opsonization effect of the reticuloendothelial system for nanoparticle sizes around 3 nm [55]. In our study, the nanoemulsion size was controlled at 17 nm, which should cross the BBB more effectively for possible evasion of the opsonization of the reticuloendothelial system.

It has been established that the potential pathways involved in nanoparticle-mediated drug trafficking across the BBB associated with AD or PD include hydrophilic paracellular pathway, ligand–nanoparticle receptor-mediated transcytosis, ionized nanoparticle adsorptive transcytosis, the functionalized nanoparticle carrier-mediated pathway, and the lipophilic transcellular pathway [56]. Taking into account the nanoemulsion characteristics prepared in our study, a combination of various pathways may be involved in the crossing of the BBB. Namely, in addition to hydrophilic paracellular pathway and protein-mediated transport, both adsorptive-mediated transcytosis and transcytosis (receptor-mediated and non-receptor mediated) may occur, with the former involving the adsorption of nanoemulsion onto the endothelial surface for internalization and transport across the BBB, and the latter involving the uptake of the nanoemulsion by endothelial cells, where the nano-droplets are engulfed by cell membrane. Additionally, BBB crossing may also involve passive diffusion, which is a specific type of hydrophilic paracellular pathway, and non-receptor mediated transcytosis.

Regarding the various signal pathways associated with the progress of AD, Wang et al. [2] pointed out that ROS directly affect targets such as NF-kB, TNF, PI3K, Akt, GSk-3β, and Tau, leading to mitochondrial dysfunction and neuron cell death. Additionally, ROS can indirectly affect the Wnt signaling pathway, playing a critical role, along with Wnt ligands, in mediating both intercellular and intracellular signaling through interactions with a variety of receptors and related molecules for the regulation of synaptogenesis, with Wnt directly affecting synapse formation by controlling the assembly of pre- and postsynaptic components in both central and peripheral synapses [2]. Moreover, CA was found to be efficient in retarding AD progress by raising the phosphorylated forms of Akt, ERK, and GSk-3β in the hippocampus, with PI3K-Akt signaling being a key pathway inhibiting GSk-3β by phosphorylating the serine, which, in turn, decreased tau protein phosphorylation [4]. In addition, the Akt/mTOR signaling pathways involved in protein synthesis at the synapse are essential to synaptic plasticity and memory formation, and the cognitive decline may result from an impaired Akt/mTOR pathway in AD [57]. Therefore, in the early stages of AD, CA may help to protect neurons and ameliorate cognitive decline via the activation of the PI3K/Akt signaling pathway and the inhibition of mTOR hyperactivation.

As for the signal pathways connected to PD progress, the PI3K/Akt pathway was activated to facilitate the survival and growth of dopamine neurons by inhibiting apoptosis through the downregulation of GSK-3β, which widely expressed in the central nervous system but abnormally in PD [58,59]. More importantly, GSK-3β is a negative regulator of endoplasmic reticulum stress, mitochondrial function, inflammation, glucose homeostasis, and apoptotic pathways, with Akt and phosphorylated Akt being shown to be reduced in the substantia nigra compacta of PD patients [50,60]. Furthermore, the inhibition of the PI3K-Akt/mTOR pathway has been shown to result in a decline in the expression of JNK3, a protein regulating programmed cell death and mainly present in neurons in the substantia nigra pars compacta, leading to dopaminergic protection and PD improvement [61], while the activation of the PI3K-Akt/mTOR pathway was found to impair the autophagy function associated with PD by triggering of inflammasome [62]. Thus, it is possible to treat PD and AD simultaneously through the activation of the PI3K/Akt pathway and the inhibition of the PI3K-Akt/mTOR pathway by CA, the dominant bioactive compound in CLE and CLEN. Nevertheless, for PD or AD, some other pathways, such as the HO-1 and MAPK pathways, the PLCr pathway, the NLRP3/caspase-1/gasdermin D pathway, and the BDNF/TrkB/cyclic AMP response element binding protein pathway, may also be involved in the protection of synaptic plasticity and the decrease in α-synuclein accumulation by CA, but this needs further investigation. Additionally, several recent studies have demonstrated that both insulin resistance and insulin deficiency in the brain are critical factors affecting memory loss, as evident by a decline in the levels of insulin, insulin receptor mRNA, and insulin receptor substrate, all of which are related to the PI3K/Akt pathway, being shown in animals with AD or PD [50,63]. As CA has been reported to improve type II diabetes and protect neurons from damage through the activation of the PI3K/Akt and MAPK/ERK pathways [50], it is feasible to treat type II diabetes-induced PD and AD at the same time through the intake of CLE or CLEN.

Many natural compounds, including flavonoids and non-flavonoids, such as resveratrol and curcumin, have been shown to be effective in alleviating PD and AD symptoms through the activation of the PI3K/Akt pathway [58]. However, these compounds are often eliminated rapidly in vivo because of their lipophilic nature and low bioavailability. Moreover, in many trials, a single compound was used as a dietary supplement, which may limit its therapeutic efficiency in AD and PD [58]. Conversely, in our study, the cinnamon extract containing various bioactive compounds, including CA, cinnamic acid, cinnamyl alcohol, and eugenol, were encapsulated into a nanoemulsion system, which should enhance the therapeutic effect for AD and PD through a synergistic effect and bioavailability elevation. This finding further demonstrates the potential of applying the diverse bioactive compounds in CLEN to multiple targets, overcoming the failure encountered by a single-target drug discovery strategy.

It has been demonstrated that microglial cells can cause inflammation through the activation of various cytokines [64]. Following the administration to mice of CA or TCA, a reduction in proinflammatory cytokines, including NO, IL-1β, and TNF-α, as well as the inhibition of the NF-kβ pathway, was observed [65,66]. Furthermore, the elevation of these cytokine levels has been shown to be imperative in insulin signaling impairment via the inhibition of the serine phosphorylation of insulin receptor signaling [67]. In a study dealing with the effect of TCA on LPS-induced neuroinflammation in mice, TCA was shown to significantly ameliorate the LPS-induced impairment of learning and memory, neuroinflammation, oxidative stress, and neuronal apoptosis through the activation of the nuclear factor erythroid 2-related factor 2 (Nrf2), the elevation of SOD and glutathione-S-transferase, and the reduction in interleukin-1β (IL-1β), MDA. and caspase-3 contents in the hippocampi of mice [68]. In a transgenic mice model, the oral administration of cinnamon extract was found to reduce 56 kDa Aß oligomers and plaques, improving cognitive behavior, and suggesting that the cinnamon extract possesses an advantage over drugs containing only a single component; thus, it can be developed as a therapeutic for AD treatment [69]. Similarly, the CA pretreatment at a dose of 100 mg/kg was shown to significantly prevent the amnesic effect of scopolamine-induced dysregulations of hippocampal MAPK and Akt in male mice, revealing the potential of CA to be developed as a prophylactic drug [46]. Recently Qian et al. [70] identified 30 compounds in the aqueous extract of cinnamon, in which 17 compounds had a good absorption due to the BBB limitation, with GABRA1, GABRB2, GABRA5, and GABRG2 being identified as core therapeutic targets of cinnamon against AD-related GABAergic synaptic dysfunction, and methyl cinnamate, propyl cinnamate, (+)-procyandin B2, procyandin B1, and myristicin being identified as the brain synapse-targeting active substances of cinnamon using a systematic strategy. However, this study fails to identify CA, the dominant functional compound in cinnamon, mainly because of the poor water solubility of CA. There is also an additional drawback in this study: no in vivo experiment was carried out to verify the improvement of AD through the administration of the cinnamon aqueous extract.

In a review article dealing with AD treatment with cinnamon, Momtaz et al. [71] concluded that cinnamon extract was effective in inhibiting tau accumulation, Aβ aggregation, and toxicity in both in vivo and in vitro models through the interference of multiple oxidative stress and proinflammatory pathways, as well as the modulation of endothelial functions and the attenuation of vascular cell adhesion molecules. Similarly, in another review article dealing with the neuroprotective potential of cinnamon and its metabolites in PD, Angelopoulou [72] concluded that the oral administration of cinnamon powder and sodium benzoate can be protective against motor deficits, striatal neurotransmitter dysregulation, and dopaminergic cell death through antioxidant effects, autophagy regulation, the inhibition of excessive proinflammatory cytokines, the modulation of the TLR/NF-κB pathway, the upregulation of Parkin, DJ-1, and glial cell-derived neurotrophic factor, and the retardation of α-synuclein aggregation. Both reports provide strong evidence of the possible treatment of AD and PD by cinnamon extract and its metabolite sodium benzoate. Regarding the safety issues pertaining to cinnamon, cinnamon is generally recognized as a safe food additive according to the United States Food and Drug Administration (USFDA), and no adverse reports have been shown in any of the human studies involving cinnamon and its aqueous extracts [71]. For CA, it is approved as a safe natural ingredient, with a daily intake of 1.25 mg/kg/bw, by both the USFDA and the European Council. In a recent study dealing with the preparation of a CLEN containing CA at 10,000 ppm, the CLEN was shown to ameliorate type II diabetes in rats while elevating liver, kidney, and cardiovascular functions, demonstrating the safety of CA when utilized as a nutraceutical or botanic drug [14]. Thus, cinnamon, and especially CA, should be effective and safe for use in the prevention and treatment of AD and PD. Nevertheless, further studies are required to investigate the safety of the long-term use of CA at high doses.

Although the possible prevention and treatment of AD and PD in rats by cinnamon-derived products was demonstrated in this study, there are several limitations that need to be addressed. First, the possible signal pathways responsible for AD and PD progression need to be further explored. Second, some more biochemical parameters, such as tau protein expression for AD and the levels of α-synuclein and tyrosine hydroxylase for PD, need to be measured. Third, a rat model was used in this study, which could be inadequate. A future clinical trial is necessary to verify the findings observed in this study. Most importantly, future studies should focus on identifying the common pathways involved in AD and PD for the possible development of a dual therapy involving the administration of drugs or CLEN.

## 4. Conclusions

Based on the above results indicating the possible prevention and treatment of AD and PD by CLEN and its byproducts using a rat model, the following conclusions can be made. We observed a significant amelioration of AD in rats via administration with CLEN, which can be attributed to the synergistic effect provided by a combination of functional compounds possessing antioxidant activity, such as CA, cinnamic acid, eugenol, benzoic acid, and cinnamyl alcohol in cinnamons, as well as fatty acids in soybean oil, lecithin, and Tween 80 in the nanoemulsion. As AD and PD represent a complex symptom, no single drug can be used to cure patients with AD and PD. Instead, the selection and combination of antioxidants, neurotransmitters, and anti-inflammation agents at optimal doses may, in the future, be used to treat patients in the early stages of AD and PD. Additionally, the intake of CLEN may be effective in ameliorating the symptoms of patients with PD, as evident in a substantial increase in dopamine content in the brain striatum of rats observed in this study. More importantly, in this study, we demonstrated that AD occurrence can be accompanied by PD development. PH and PW may have preventive effects against AD and PD, as a significant improvement in the long-term and short-term memory of rats; a reduction in Aβ40, BACE1, AchE, and 8-oxodG; and an increase in the antioxidant enzymes and dopamine in rat brains have been shown following pre-feeding with both samples for 4 weeks, demonstrating the potential of PH and PW to be utilized in AD- and PD-preventive medicine. Nevertheless, more clinical trials are needed in order to verify treatment efficiency through the intake of CLEN by patients with AD and PD.

## Figures and Tables

**Table 1:** Identification and quantitation data of functional compounds in cinnamon leaf powder and hydrosol by UPLC-MS/MS.

Peak	Compound ^a^	Retention Time (min)	MS/MS (*m*/*z*)		Content (μg/g) ^c^	
			**Precursor Ion**	**Product Ion**	**Powder**	**Hydrosol ^e^**
1	5-O-Caffeoylquinic acid	2.51	353	191	0.4 ± 0.1	ND ^d^
2	Caffeic acid	4.38	179	134	2.4 ± 0.1	ND
3	p-Coumaric acid	5.77	164	90	4.7 ± 0.8	ND
4	Coumarin	5.78	147	91	2.6 ± 0.2	ND
5	Benzoic acid	6.83	121	77	67.7 ± 0.6	4.2 ± 0.3
6	Rutin	7.46	609	300	5.0 ± 0.2	ND
7 ^b^	Quercetin-3-O-galactoside	7.51	463	300	5.5 ± 0.1	ND
8 ^b^	Quercetin-3-O-glucoside	7.51	463	300	-	ND
9	Quercetin	7.54	301	151	20.9 ± 1.8	ND
10	Cinnamyl alcohol	8.47	117	115	92.2 ± 5.2	4.0 ± 0.4
11	Cinnamaldehyde	8.65	132	55	18,250.7 ± 334.86	1218.8 ± 92.4
12	Kaempferol	8.73	287	153	4.9 ± 06	ND
13	Kaempferol-3-β-D-glucopyranoside	8.62	447	284	19.9 ± 0.4	ND
14	trans-Cinnamic acid	9.39	147	103	464.9 ± 7.8	1.94 ± 0.11
15	Eugenol	11.05	165	137	220.1 ± 1.9	14.5 ± 0.2

^a^ Peaks were positively identified based on the comparison of the retention time and mass spectra of the standards with unknown peaks. ^b^ Both peaks overlapped, with the content being 5.5 ± 0.1 μg/g (quercetin-3-O-galactoside plus quercetin-3-O-glucoside). ^c^ Data are presented as the mean ± standard deviation of triplicate determinations. ^d^ Not detected. ^e^ Directly analyzed by UPLC-MS/MS without solvent extraction.

**Table 2:** The average particle size, polydispersity index, and zeta-potential changes in CLEN during storage at 4 °C for 90 days.

Day	Particle Size (nm) ^a,b^	Polydispersity Index (PDI) ^a,b^	Zeta Potential (mV) ^a,b^
0	17.3 ± 1.5 ^B^	0.177 ± 0.088 ^A^	−42.1 ± 0.9 ^A^
15	16.9 ± 3.0 ^B^	0.160 ± 0.074 ^A^	−42.8 ± 1.9 ^A^
30	19.1 ± 1.4 ^AB^	0.102 ± 0.047 ^A^	−41.2 ± 2.0 ^A^
45	19.7 ± 2.1 ^AB^	0.110 ± 0.042 ^A^	−41.9 ± 2.6 ^A^
60	20.7 ± 2.9 ^A^	0.063 ± 0.073 ^A^	−41.0 ± 1.2 ^A^
75	22.1 ± 2.2 ^A^	0.099 ± 0.042 ^A^	−39.6 ± 2.6 ^A^
90	23.2 ± 1.6 ^A^	0.169 ± 0.106 ^A^	−40.0 ± 0.9 ^A^

^a^ Data shown as mean ± standard deviation (*n* = 3). ^b^ Data with different capital letters (^A,^ ^B^) in a column are significantly different at *p* < 0.05.

## Data Availability

The original contributions presented in this study are included in the article and Supplementary Materials. Further inquiries can be directed to the corresponding authors.

## Supplementary Materials

The following supporting information can be downloaded at: https://www.mdpi.com/article/10.3390/pharmaceutics17091200/s1, Calibration curves for 15 functional compounds quantitifed in cinnamon leaf powder and hydrosol.

## Abbreviations

AchE	acetylcholinesterase
AD	Alzheimer’s disease
Akt	serine/threonine-specific protein kinase
ANOVA	analysis of variance
ApoE	apolipoprotein E
ATP	adenosine triphosphate
Aβ40	amyloid beta protein 40
BACE1	beta-site amyloid precursor protein cleaving enzyme 1 (β-secretase 1)
BBB	blood–brain barrier
BCA	bicinchoninic acid
BDNF	brain-derived neurotrophic factor
BuChE	butyrylcholinesterase
bw	body weight
CA	cinnamaldehyde
cAMP	cyclic adenosine monophosphate
CLE	cinnamon leaf extract
CLEN	cinnamon leaf extract nanoemulsion
CAT	catalase
CNS	Chinese National Standard
DHA	docosahexaenoic acid
DJ-1	parkinsonian disease protein 7/parkinsonism-associated deglycase
DLS	dynamic light scattering
DNA	deoxyribonucleic acid
EPA	eicosapentaenoic acid
ERK	extracellular signal-regulated kinase
ESI	electrospray ionization
GABRA1	gamma aminobutyric acid A receptor alpha 1
GABRA5	gamma aminobutyric acid A receptor alpha 5
GABRB2	gamma aminobutyric acid A receptor beta 2
GABRG2	gamma aminobutyric acid A receptor gamma 2
GLUT1	glucose transporter type 1
GSH-Px	glutathione peroxidase
GSK-3β	glycogen synthase kinase 3 beta
HE	high-dose extract
HLB	hydrophilic–lipophilic balance
HN	high-dose cinnamon leaf extract nanoemulsion
HPLC	high-performance liquid chromatography
HRP	horseradish peroxidase
IACUC	Institutional Animal Care and Use Committee
IL-1β	interleukin 1 beta
IRS-1	insulin resistance substrate 1
LDL	low-density lipoprotein
LE	low-dose extract
LN	low-dose cinnamon leaf extract nanoemulsion
LPS	lipopolysaccharide
MAO	monoamine oxidase
MAPK	mitogen-activated protein kinase
MDA	malondialdehyde
ME	medium-dose extract
MN	medium-dose cinnamon leaf extract nanoemulsion
MRM	multiple reaction monitoring
N	normal/control group
NF-κB	nuclear factor kappa beta
NO	nitric oxide
Nrf2	nuclear factor erythroid 2-related factor 2
NT-3	neurotrophin 3
8-oxodG	8-hydroxy-2′-deoxyguanosine
PBS	phosphate-buffered saline
PD	Parkinson’s disease
PDI	polydispersity index
PH	cinnamon powder in hydrosol
PI3K	phosphoinositide 3-kinase
PKA	protein kinase A
PW	cinnamon powder in water
ROS	reactive oxygen species
RSD	relative standard deviation
SABC	horseradish–streptavidin biotin conjugate
SOD	superoxide dismutase
STZ	streptozotocin
Tau	tubulin-associated unit protein
TCA	trans-cinnamaldehyde
TEM	transmission electron microscopy
TMB	3,3′,5,5′-tetramethylbenzidine
TNF-α	tumor necrosis factor alpha
UPLC-MS/MS	ultra-performance liquid chromatography–tandem mass spectrometry
USFDA	United States Food and Drug Administration
UV	ultraviolet
Wnt	wingless-related integration site
5XFAD	5 familial Alzheimer’s disease mutation mice model
