# Supplementary material for: Improving Alzheimer’s Disease and Parkinson’s Disease in Rats with Nanoemulsion and Byproducts Prepared from Cinnamon Leaves
Source: Pharmaceutics. 2025 Sep 15;17(9):1200. doi: 10.3390/pharmaceutics17091200 (PMC12473313; doi:10.3390/pharmaceutics17091200)
Supplement: Supplementary file 1 [file pharmaceutics-17-01200-s001.zip › pharmaceutics-3830608-supplementary.pdf]

Supporting Information for

Improving Alzheimer's Disease and Parkinson's Disease in Rats with Nanoemulsion and Byproducts Prepared from Cinnamon Leaves

Bing-Huei Chen [1,*], Chen-Te Jen [2], Chia-Chuan Wang [3] and Min-Hsiung Pan [2,*]

[1] Department of Food Science, Fu Jen Catholic University, New Taipei City 242062, Taiwan
[2] Graduate Institute of Food Science, National Taiwan University, Taipei City 106319, Taiwan; d11641002@ntu.edu.tw
[3] School of Medicine, College of Medicine, Fu Jen Catholic University, New Taipei City 242062, Taiwan; 050814@mail.fju.edu.tw
* Correspondence: 002622@mail.fju.edu.tw (B.-H.C.); mhpan@ntu.edu.tw (M.-H.P.)

Calibration curves for 15 functional compounds quantitifed in cinnamon leaf powder and hydrosol

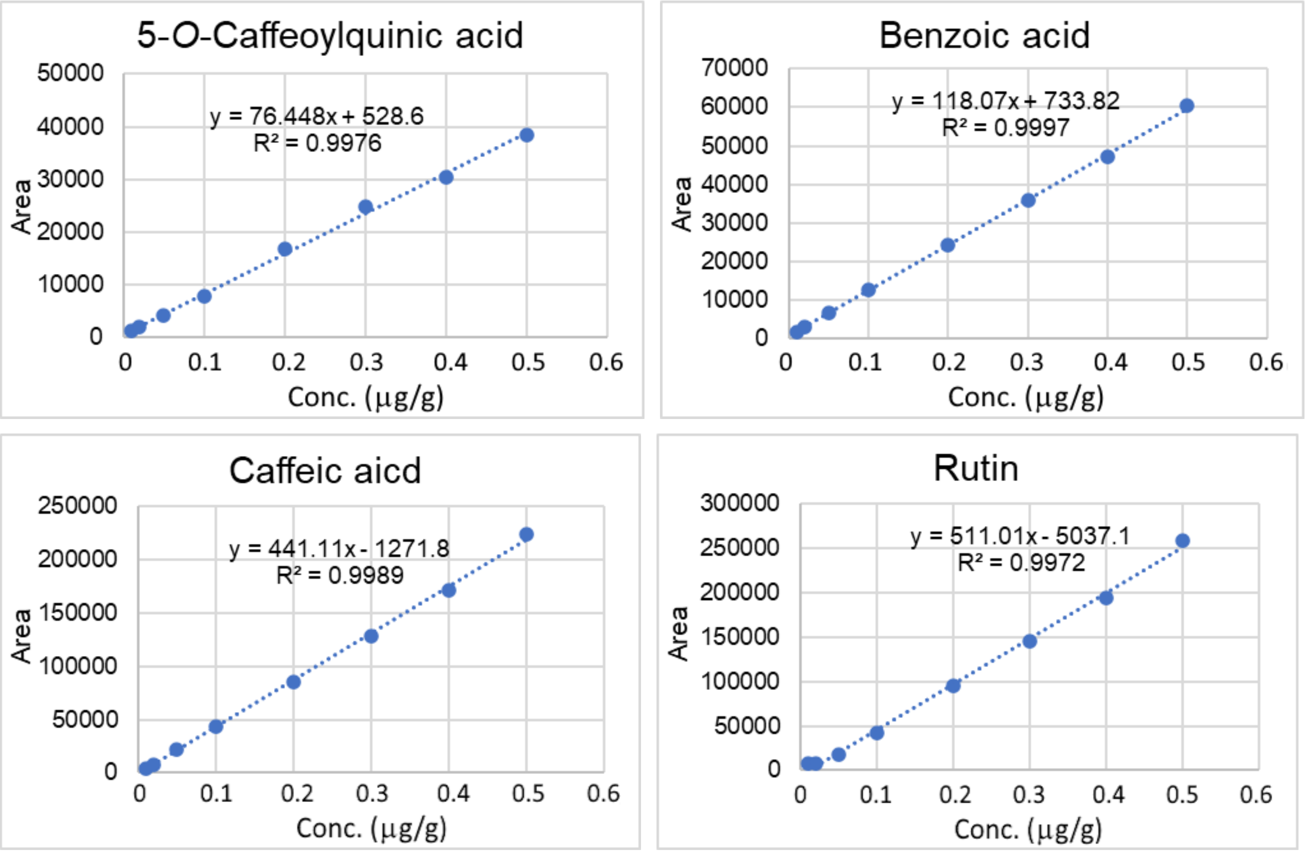

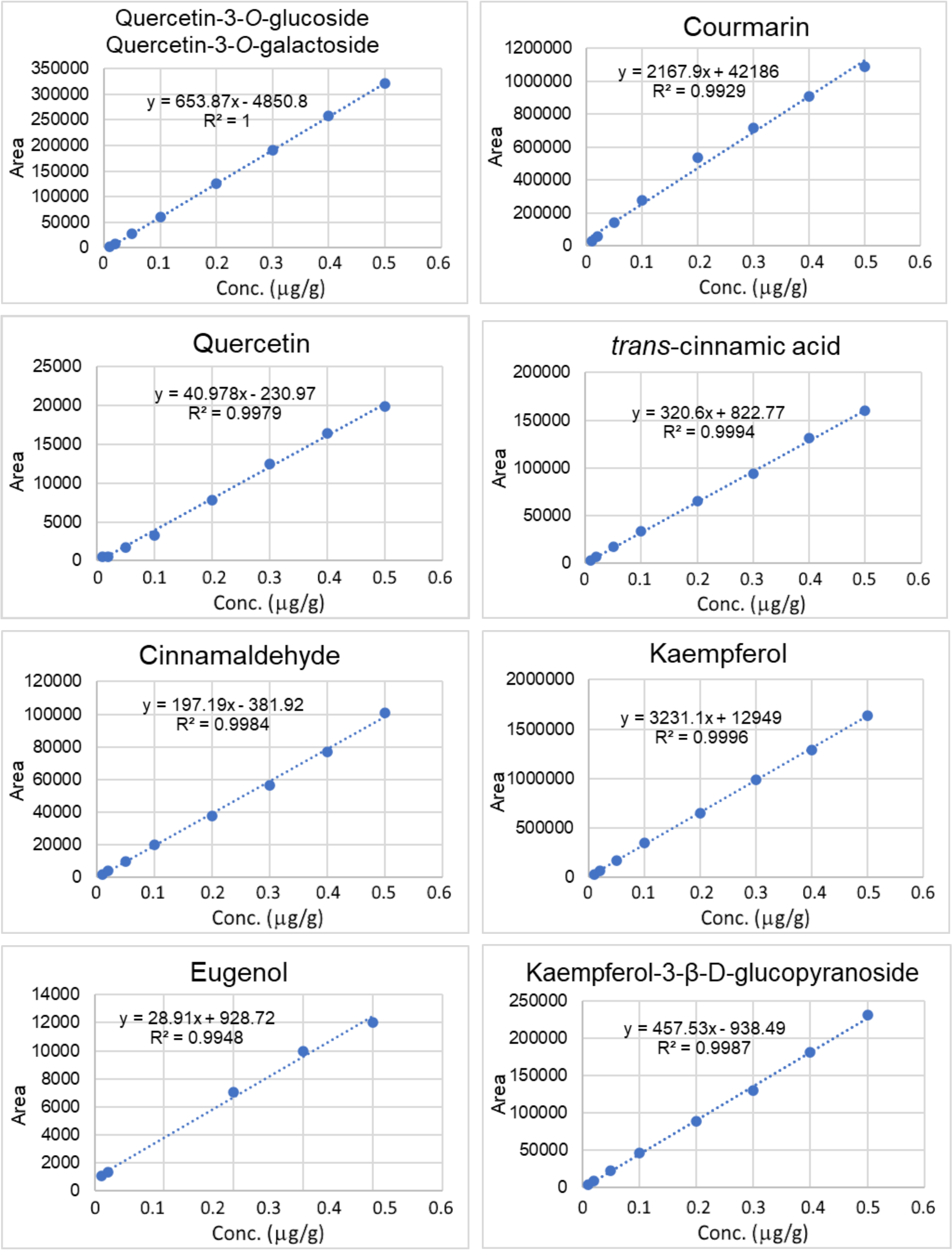
Quercetin-3-O-glucoside
Quercetin-3-O-galactoside
y = 653.87x - 4850.8
R² = 1
Area
Conc. (µg/g)
Courmarin
y = 2167.9x + 42186
R² = 0.9929
Area
Conc. (µg/g)
Quercetin
y = 40.978x - 230.97
R² = 0.9979
Area
Conc. (µg/g)
trans-cinnamic acid
y = 320.6x + 822.77
R² = 0.9994
Area
Conc. (µg/g)
Cinnamaldehyde
y = 197.19x - 381.92
R² = 0.9984
Area
Conc. (µg/g)
Kaempferol
y = 3231.1x + 12949
R² = 0.9996
Area
Conc. (µg/g)
Eugenol
y = 28.91x + 928.72
R² = 0.9948
Area
Conc. (µg/g)
Kaempferol-3-β-D-glucopyranoside
y = 457.53x - 938.49
R² = 0.9987
Area
Conc. (µg/g)

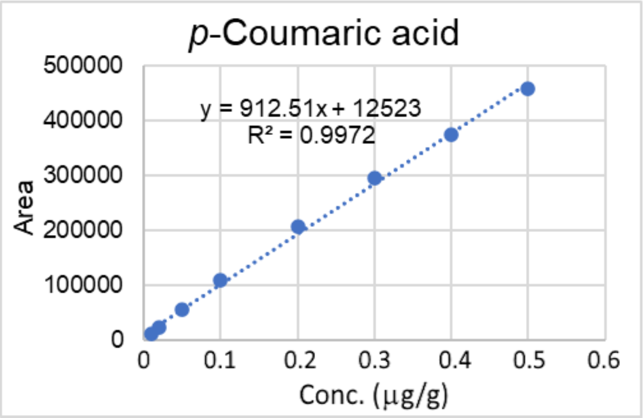
p-Coumaric acid
y = 912.51x + 12523
R² = 0.9972
Area
Conc. (µg/g)
500000
400000
300000
200000
100000
0
0
0.1
0.2
0.3
0.4
0.5
0.6

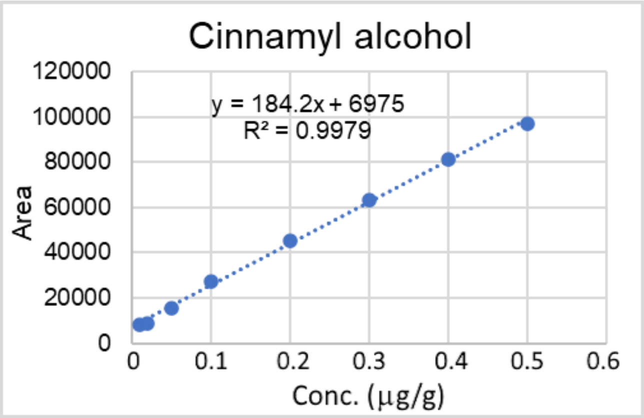
Cinnamyl alcohol
y = 184.2x + 6975
R² = 0.9979
Area
Conc. (µg/g)
120000
100000
80000
60000
40000
20000
0
0
0.1
0.2
0.3
0.4
0.5
0.6
